# Advancing insights into in vivo meningeal lymphatic vessels with stereoscopic wide-field photoacoustic microscopy

**DOI:** 10.1038/s41377-024-01450-0

**Published:** 2024-04-25

**Authors:** Fei Yang, Zhiyang Wang, Wenbin Shi, Miao Wang, Rui Ma, Wuyu Zhang, Xipeng Li, Erqi Wang, Wenjie Xie, Zhan Zhang, Qi Shen, Feifan Zhou, Sihua Yang

**Affiliations:** 1 MOE Key Laboratory of Laser Life Science & Institute of Laser Life Science, South China Normal University, Guangzhou, 510631 China; 2 Guangdong Provincial Key Laboratory of Laser Life Science, College of Biophotonics, South China Normal University, Guangzhou, 510631 China; 3 Key Laboratory of Biomedical Engineering of Hainan Province, School of Biomedical Engineering, Hainan University, Haikou, 570100 China; 4 Department of Neurology, Sun Yat-sen Memorial Hospital, Sun Yat-sen University, Guangzhou, 510120 China; 5 Guangdong Province Key Laboratory of Brain Function and Disease, Zhongshan School of Medicine, Sun Yat-sen University, Guangzhou, 510120 China; 6 Guangdong Basic Research Center of Excellence for Structure and Fundamental Interactions of Matter, South China Normal University, Guangzhou, 510006 China

**Keywords:** Photoacoustics, Biophotonics

## Abstract

Meningeal lymphatic vessels (mLVs) play a pivotal role in regulating metabolic waste from cerebrospinal fluid (CSF). However, the current limitations in field of view and resolution of existing imaging techniques impede understanding the stereoscopic morphology and dynamic behavior of mLVs in vivo. Here, we utilized dual-contrast functional photoacoustic microscopy to achieve wide-field intravital imaging of the lymphatic system, including mLVs and glymphatic pathways. The stereoscopic photoacoustic microscopy based on opto-acoustic confocal features has a depth imaging capability of 3.75 mm, facilitating differentiation between mLVs on the meninges and glymphatic pathways within the brain parenchyma. Subsequently, using this imaging technique, we were able to visualize the dynamic drainage of mLVs and identify a peak drainage period occurring around 20–40 min after injection, along with determining the flow direction from CSF to lymph nodes. Inspiringly, in the Alzheimer’s disease (AD) mouse model, we observed that AD mice exhibit a ~ 70% reduction in drainage volume of mLVs compared to wild-type mice. With the development of AD, there is be continued decline in mLVs drainage volume. This finding clearly demonstrates that the AD mouse model has impaired CSF drainage. Our study opens up a horizon for understanding the brain’s drainage mechanism and dissecting mLVs-associated neurological disorders.

## Abstract (web summary)

The stereoscopic photoacoustic microscopy is proposed to achieve wide-field intravital imaging of the lymphatic system in mice, facilitating three-dimensional high-resolution visualization of meningeal lymphatic vessels on the meninges.

## Graphical abstract

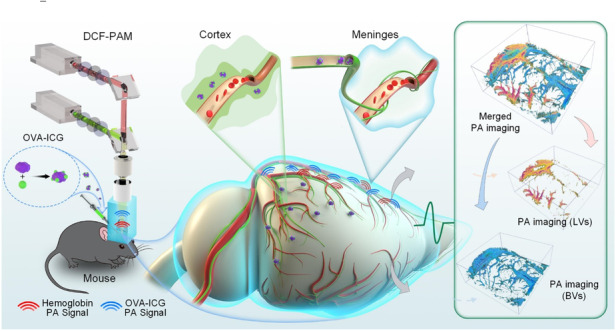

## Introduction

The central nervous system (CNS) has long been considered an ‘immune privilege’ organ lacking lymphatic vessels that transport immune cells^1–3^. However, recent studies on intracranial clearance have revealed the presence of meningeal lymphatic vessels (mLVs) and the glymphatic pathway for interstitial fluids and cerebrospinal fluid (CSF) drainage^4–8^. The mLVs continuously drain macromolecules, wastes, and superfluous liquids from the CNS to the periphery by connecting deep cervical lymph nodes (cLNs) and play an important role in the active transport of immune cells^9–11^. Among them, permeable collecting lymphatics and capillary lymphatics are distributed along the sigmoid sinus and petrosquamous sinus of the skull base. The lymphatic vessels located in the meninges are distributed along the transverse sinus (TS) and superior sagittal sinus (SSS)^12–15^. It is important to identify and characterize the routes responsible for CNS lymphatic drainage because paths of these may offer a valuable approach to the treatment of CNS-associated neuroinflammatory conditions.

Disruption of the mLVs and cerebral vessels (CVs) have been reported to occur and contribute to the disease progression during aging as well as in neurodegenerative and neurological diseases, such as Alzheimer’s disease (AD)^16–19^, Parkinson’s disease^20^, traumatic brain injury^21–23^, encephalitis^24,25^, and brain tumor^26,27^. Differences in the morphology and function of mLVs and CVs based on sex and age may also be related to sex and age differences in the incidence of certain neurological diseases^28–30^. Retrieving a whole-brain perspective of mLVs and CVs with sufficient resolution can enhance understanding of the complexity of brain immune function. A more comprehensive description of lymphatic and vascular systems can effectively illustrate the immune function of the brain and how the lymphatic and vascular system contributes to the pathogenesis of various diseases, involving immune and inflammatory responses and their respective role. Therefore, there is a need for an intravital imaging method capable of simultaneously visualizing mLVs and CVs with high temporal and spatial resolution to gain insight into immune function of the brain.

Previous studies have observed these structures through the use of electron microscopy, histology, immunofluorescence (IF), and nuclear magnetic resonance imaging (MRI). IF with the merit of excellent resolution has been widely utilized to assess the structure and function of mLVs and CVs in various fields^22,26^. However, it requires staining and imaging in vitro, which limits its application for dynamic in vivo monitoring. Similarly, high Tesla MRI is available for brain imaging in living animals^31,32^. In a few studies, the depiction of CSF from mLVs to cLNs has been achieved in the human brain^33,34^. MRI allows for in vivo macroscopic evaluation of CVs and mLVs using intravenous and intrathecal Gadolinium (Gd)^15^. It is inevitable that even the spatial resolution of high Tesla MRI is still insufficient for imaging structural information of mLVs in mice. The use of a stereo microscope can quantify the transport of tracers to the blood and lymph nodes^29^, but it is unable to distinguish mLVs from the results. Multiphoton microscopy can also capture the dynamics of mLVs and CVs. Intravital two-photon microscope approaches with various probes have enabled the monitoring of CSF tracers at the microscopic scale^12,30^. It’s important to note that these methods often involve craniotomy, which can disrupt brain hemostasis or offer a limited field of view (FOV) where the tracer can only be observed in a small brain region. Therefore, there is a need for high-resolution three-dimensional simultaneous imaging of mLVs and CVs in vivo to facilitate safer and more comprehensive visualization of their structure and function, advancing research related to the drainage pathway of mLVs or CSF. As a hybrid imaging technique, photoacoustic imaging combines the advantages of optical resolution and acoustic penetration depth and has made progress in brain imaging and lymphatic imaging in recent years. Using wide-field and deep-penetration three-dimensional (3D) photoacoustic tomography with a hemispherical detector array, blood vessels and lymphatic vessels in patient’s limb could be imaged^35–37^, which showed the advantage of photoacoustic imaging in vessel structure and potential clinical application. However, the spatial resolution of this system is still limited for stereoscopic visualization of the mLVs that are tens of microns in size within the mouse brain. In addition, as a newly discovered drainage channel, the structural feature of mLVs is not clear. Given the current situation, a technical means with high spatial resolution that can specifically distinguish CVs and mLVs is needed to accurately assess the morphology and function of CVs and mLVs.

**Fig. 1:**
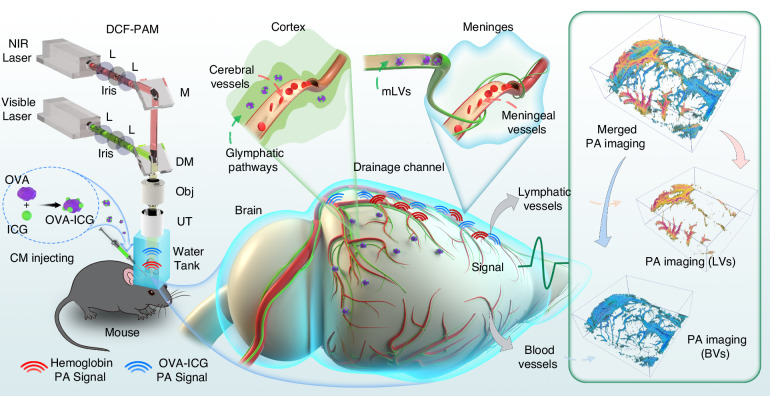
Intravital co-localized imaging of mLVs and CVs. Two wavelengths are focused on the same depth position and scanned alternately to capture images of mLVs and CVs. Exogenous contrast nanoprobes, created by loading ICG dye on OVA, are utilized to enhance the visualization of mLVs. CVs are imaged using a visible laser, relying on endogenous contrast from hemoglobin. The photoacoustic signals generated by the exogenous tracer and the endogenous hemoglobin are first coupled through a water tank filled with deionized water and then detected by the ultrasound transducer. M mirror, DM dichroic mirror, L lens; Obj, objective lens; UT, ultrasound transducer

In the article, we demonstrate intravital co-localized imaging of mLVs and CVs using a dual-contrast functional photoacoustic microscope (DCF-PAM) as shown in Fig. 1. DCF-PAM provides a lateral resolution of 8.9 μm and 6.1 μm within a FOV of 12 × 12 mm^2^. The scanning range encompasses the transverse section of the entire mouse brain. For imaging mLVs, an exogenous contrast tracer is designed by loading the dye indocyanine green (ICG) onto ovalbumin (OVA). After entering CSF, the exogenous contrast tracer (OVA-ICG) is largely drained through mLVs and glymphatic pathways. The endogenous contrast hemoglobin is used for imaging CVs. Two wavelengths are focused on the same depth position and scanned alternately to image tracers and CVs. The stereoscopic photoacoustic (PA) imaging of the tracers is effectively segmented into the mLVs and glymphatic system. The wide-field PA imaging of mLVs aligns with the structures revealed by immunofluorescence of mLVs, confirming that DCF-PAM enables dynamic monitoring of CSF drainage while maintaining high resolution. The 9.4 T MRI and PA B-scan illustrations exhibit spatial correspondence, with the latter presenting greater detail, resolution, and sensitivity. Naturally, DCF-PAM allows for long-term monitoring of live animals during testing. The time-phased recording of mLVs demonstrates the drainage process of CSF from the mLVs to the cLNs. Our data revealed that the mLVs function is remarkably dysregulated in AD mice, including disrupted drainage volume with decreased gene expression of lymphatic function. Such alterations are likely due to Aβ deposition, which consequently led to impaired CSF drainage function. Moreover, the reduced drainage volume of mLVs in young mice compared to adult mice maybe because of incomplete development of the meningeal function. Collectively, intravital co-localized imaging of mLVs and CVs is demonstrated using DCF-PAM, enabling wide-field monitoring of mLVs and CVs and capturing dynamic drainage and clearance processes in the brain. This approach holds promise as a valuable tool for studying diseases associated with mLVs, such as neuroinflammation and neurodegenerative disorders.

## Results

### Characterization of dual-contrast functional microscopy and tracer

**Fig. 2:**
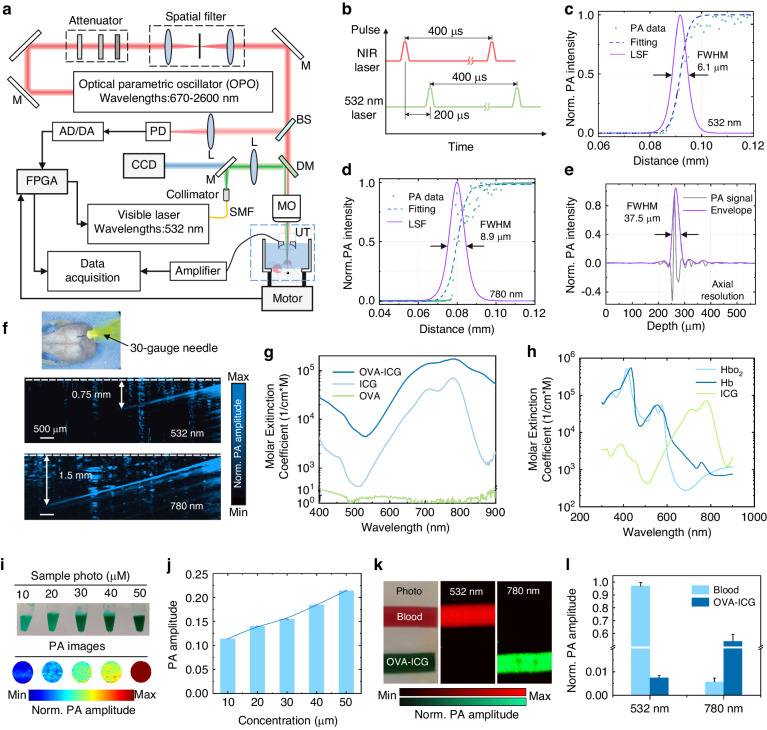
The schematic and characterization of the DCF-PAM and OVA-ICG. **a** Schematic diagram of the DCF-PAM setup. **b** All lasers fire at a frequency of 2.5 kHz, and the delay time between the dual pulse is 200 μs. **c**, **d** The lateral resolution of visible laser and NIR laser. **e** The axial resolution of the system. **f** Photograph and measured penetration depths for the visible laser and NIR laser under the intact skull. **g** VIS-NIR spectra of OVA, ICG, and OVA-ICG. **h** UV-VIS-NIR spectra of blood and ICG. **i** Sample photos and PA images of different concentrations of OVA-ICG. **j** Comparison of PA amplitudes for these concentrations of OVA-ICG. **k** Sample photos and PA images of blood and OVA-ICG using DCF-PAM. **l** Comparison of PA amplitudes of blood and OVA-ICG. M mirror, BS beam splitter, DM dichroic mirror, L lens, MO microscope objective lens, UT ultrasound transducer, SMF single-mode fiber, PD photodiode, AD/DA Analog to Digital/Digital to Analog Converter, FPGA Field Programmable Gate Array, FWHM full width at half maximum, LSF line spread function

The schematic of DCF-PAM is depicted in Fig. 2a. The near-infrared (NIR) laser, emitted by the optical parametric oscillator (NT-200, Ekapla), operates at a repetition rate of 2.5 kHz. The NIR laser undergoes spatial filtering through an attenuator and a spatial filter, followed by reflection from a dielectric mirror and transmission through a dichroic mirror. It is then combined with the visible laser. The 532-nm pulsed laser (DTL-314QT, Laser-export) is coupled to a single-mode fiber using a fiberport coupler (PAF2-7A, Thorlabs). The visible laser, collimated by a collimator (F240FC-532, Thorlabs), is reflected by the dichroic mirror and focused by a microscope objective lens (GCO-2111, Daheng). A self-focused high-frequency ultrasound transducer (central frequency: ~40 MHz, bandwidth: 21-63 MHz, focal length: 8 mm) with a ring-shaped PVDF-based piezoelectric element (3 mm inner diameter, 8 mm outer diameter), is positioned beneath the microscope lens. The laser emits from the center of the ring and irradiates the mouse brain. A two-dimensional motorized translation stage (LS2-25T, Jiancheng optics) located beneath the mouse brain is utilized for scanning. The motorized translation stage translates the mouse in the *x* and *y* directions for raster scanning. The collected PA signals are amplified by a 50-dB gain amplifier (LNA-650, RF Bay). Subsequently, the amplified PA signals are digitized by a data acquisition card (M4i.4420, Spectrum). Simultaneously, the NIR laser is split by a beam splitter, and the signal trigger is captured by a photodiode and transmitted to a field-programmable gate array (FPGA). The lasers’ triggers, the motorized transducer stage, and the collection of PA signals are all controlled by FPGA.

During dual-wavelength illumination, all lasers are fired at a frequency of 2.5 kHz, with a delay time of 200 μs between the dual-pulse (Fig. 2b). The lateral resolutions of the visible and NIR lasers are characterized by measuring a sharp blade, resulting in resolutions of 6.1 μm and 8.9 μm, respectively (Fig. 2c, d). The axial resolution of the system is estimated to be 37.5 μm (Fig. 2e). The nanoparticle (CuS) is immobilized into an agar phantom to evaluate the depth imaging capability and the spatial resolution, where light scattering is dominated by the agar (See Supplementary Text and Fig. S1–S3). The system maintained acceptable lateral resolution and signal-to-noise within a depth of 3.75 mm (Fig. S4). Further, the penetration depth through the intact skull is measured by simulating a real environment with a 30-gauge needle inserted obliquely (~15–30 degrees) into the mouse brain (Fig. S5), where photon scattering is primarily caused by the skull and brain parenchyma. The transcranial penetration depths of the visible laser and NIR laser are 0.75 mm and 1.5 mm, respectively (Fig. 2f). Since the mLVs locate on the dura mater under the skull, this depth is sufficient to cover their range for imaging. Finally, the imaging depth for multiple scattering samples is further demonstrated by tilting tungsten wires into the agar under the skull (Fig. S6).

Amphiphilic ICG effortlessly attaches to OVA through noncovalent interactions, resulting in the formation of OVA-ICG^38^. The loading of ICG endows remarkable NIR absorption to the OVA-ICG (Fig. 2g) and excellent biosafety (See Supplementary Text and Fig. S7 and S8). The mLVs have been reported to drain macromolecules and immune cells from CSF into cLNs^8^, and the OVA-ICG with a diameter of 30 nm fulfills the requirements for such drainage (Fig. S9). Consequently, specific wavelengths corresponding to the spectra of hemoglobin and ICG are selected for imaging purposes. Considering the high absorption coefficient of OVA-ICG at approximately 780 nm, images acquired at this wavelength are employed for evaluating the mLVs (Fig. 2h). The fluence levels of the 532 nm laser and 780 nm laser used in the system are 7.96 mJ cm^-2^ and 16.56 mJ cm^-2^, respectively, which below the applicable American National Standards Institute safety limits for laser exposure^39^.

PA imaging and analysis are performed using different concentrations of OVA-ICG (Fig. 2i, j, Detailed statistical information is shown in Table S1). Tracer with a concentration of 50 μM are withdrawn at 10 μL and injected into the cisterna magna (CM) of the mouse, ensuring the detectability of the tracer in the CSF even after dilution. Contrast images are obtained using the system, which provided PA images of hemoglobin and OVA-ICG (Fig. 2k). The signal amplitudes of hemoglobin at 532 nm laser and 780 nm laser are 0.97 ± 0.03 and 0.006 ± 0.002, while those of OVA-ICG are 0.007 ± 0.0008 and 0.54 ± 0.05 (Fig. 2l, data are presented as mean ± s.e.m.). Therefore, the system demonstrated effective differentiation between hemoglobin and OVA-ICG with high sensitivity. Simultaneous imaging of mLVs and CVs is feasible. Moreover, the laser energy at 780 nm is able to acquire the PA image of tracer in the presence of a blood background (Fig. S10). This indicates that DCF-PAM maintains high performance even when applied through the skull.

### Stereoscopic PA illustrations of mLVs and CVs

**Fig. 3:**
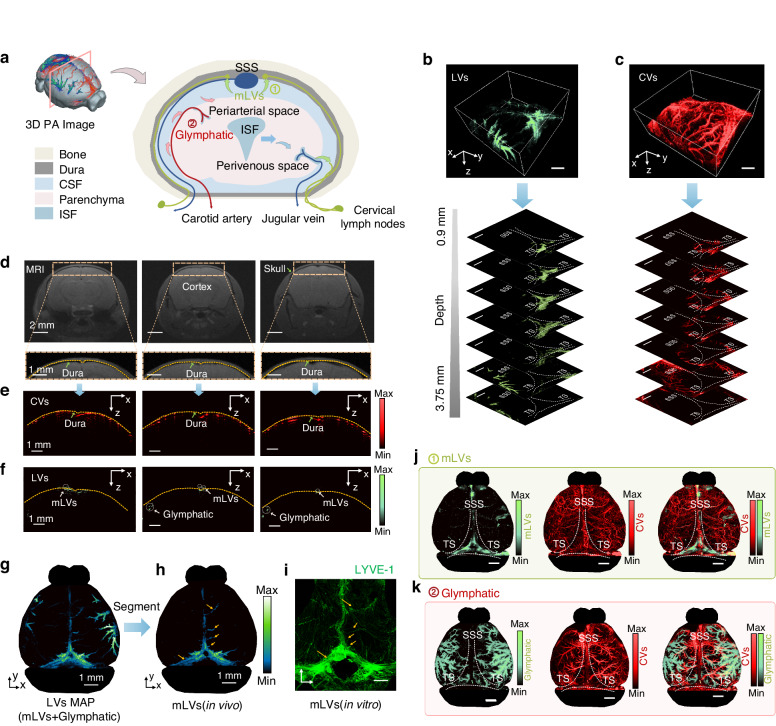
Stereoscopic PA illustrations of mLVs and CVs. **a** The 3D PA image and brain coronal plane diagram. **b**, **c** The stereoscopic PA morphology can be layered within a depth range of approximately 3.75 mm (scale bar, 1 mm). **d** The MR images of the coronal plane. **e**, **f** The PA B-scan images of CVs and LVs. **g** PA MAP images. **h** The mLVs are segmented in the PA image. **i** In vitro fluorescence imaging of the meninges. **j** The PA MAP images of mLVs and CVs. **k** The PA MAP images of glymphatic and CVs. ISF interstitial fluid, MAP maximum projection

The tracer injected into the CM in mice flows into mLVs and glymphatic pathways, participating in the drainage of interstitial fluids and CSF. The mLVs play a continuous role in removing macromolecules, waste products, and excess fluid from the CNS by connecting with deep cLNs (Fig. S11). The 3D morphology with a depth of 3.75 mm of mLVs, glymphatic pathways, and cerebral vessels is obtained using DCF-PAM (Fig. 3a–c, Movie. S1). Using the assistance of 9.4 T MRI image, the outline of the brain skull, dura mater, and brain parenchyma are delineated (Fig. 3d). Then, the reference lines are manually aligned with the PA tomography images of CVs through the optimal contrast provided for the structure of the cortical vessels of the brain (Fig. 3e). The same transformation matrix is then applied to the PA tomography images of LVs and manual segmentation is performed (Fig. 3f). Some mLVs segmented in the PA image exhibit spatial correspondence with in vitro fluorescence imaging of the meninges (Fig. 3g–i, orange arrow), confirming the consistency or alignment between the identified structures or features observed in both imaging modalities. So far, DCF-PAM enables differentiation between mLVs located on the meninges and glymphatic pathways situated within the brain parenchyma (Fig. 3j, k), providing 3D insights into the distinct anatomical locations of these lymphatic structures.

### PA spatial distribution validation of mLVs

**Fig. 4:**
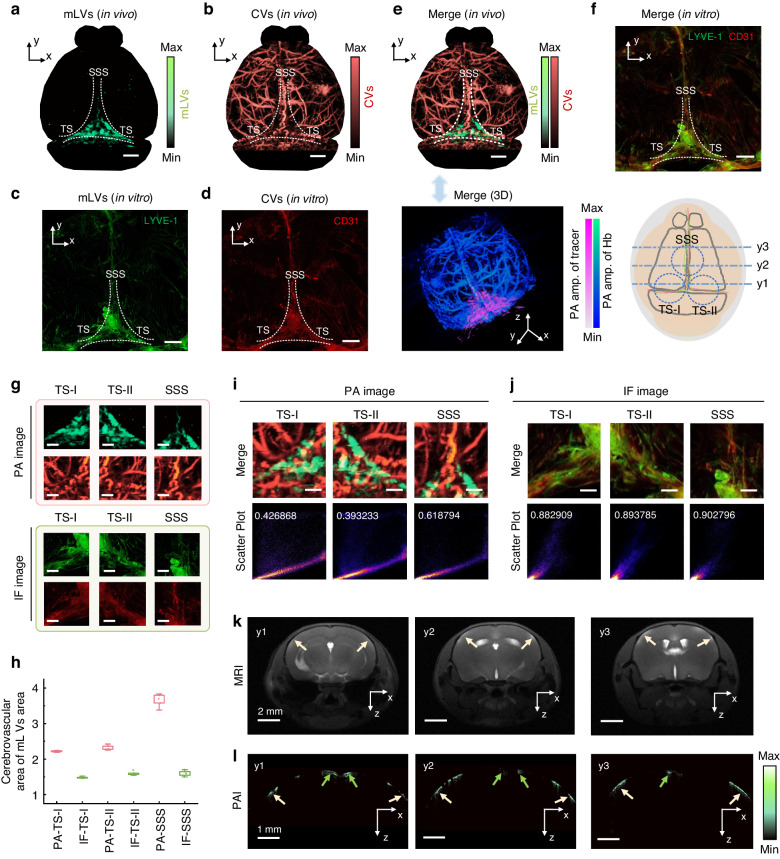
PA spatial distribution validation of mLVs. **a**, **b** PA image showing the spatial distribution of mLVs and CVs (scale bar, 1 mm). **c**, **d** IF images displaying the spatial distribution of mLVs and CVs (scale bar, 1 mm). **e**, **f** Merged PA and IF images. **g** Various images selected for the specific regions, including the near TS region and SSS region (scale bar, 1 mm). **h** Ratio of CVs area to mLVs area. **i**, **j** Merged images of the mLVs and CV divided into three regions, along with co-localization analysis. **k** MR images of the coronal plane. **l** Maximum amplitude projection in the X-Z cross-sectional direction for 10 consecutive B-scan images near y1, y2, and y3 regions, respectively

Each of the PA and IF images come from the same mouse, ensuring direct comparison between the two imaging modalities that are acquired (Fig. 4a–d). Both PA and IF imaging of mLVs exhibit similar patterns, with signals primary distributed in the TS and SSS regions. CV signals are present in both imaging modalities, but they appear more prominent and brighter in the PA rendering. In fluorescence imaging, only CV structures on the meninges are captured, while PA imaging provides a view of multilayered 3D structures. The merged PA and IF images show close resemblance (Fig. 4e, f). These merged images highlight the spatial distribution of PA imaging more clearly. Specifically, region-specific images (Fig. 4g) are selected, focusing on the TS and SSS regions. The ratio of PA modality in TS region and SSS region are ~1.5- and ~2.3-fold higher than those of IF modality, respectively. This is due to the CVs in the PA modality exhibiting a signal covering the entire living brain tissue, which exceeds the signal areas shown in the IF modality, resulting in a higher ratio (Fig. 4h and Table S2). Analyze the spatial overlap of mLVs and CVs in the meninges’ same structure and determine the positional relationship of the two structures in the meninges and possible interactions. With the co-localized overlap of signals being slightly lower in the merged PA images (Fig. 4i) compared to the merged IF images and ~half that of merged IF images (Fig. 4j and Fig. S12). This suggests that DCF-PAM provides more detailed information on the spatial distribution of mLVs and CVs.

Compared with MRI images, PA images can more clearly reflect the location distribution of mLVs. We selected 10 consecutive B-scan images near y1, y2, and y3 regions respectively, and performed maximum amplitude projection in the X-Z cross-sectional direction (Fig. 4l), and then compared them with the T1-weighted MR images acquired after injection of Gd (Fig. 4k). The observed PA signal gain (green arrow) in the brain cortex region is significantly higher than the observed MRI signal gain, further highlighting the superior contrast and resolution of mLVs in the PA image. This demonstrates that DCF-PAM provides a clearer visualization of mLVs, offering a more detailed and stereoscopic spatial distribution.

### PA insights on drainage and clearance of mLVs

**Fig. 5:**
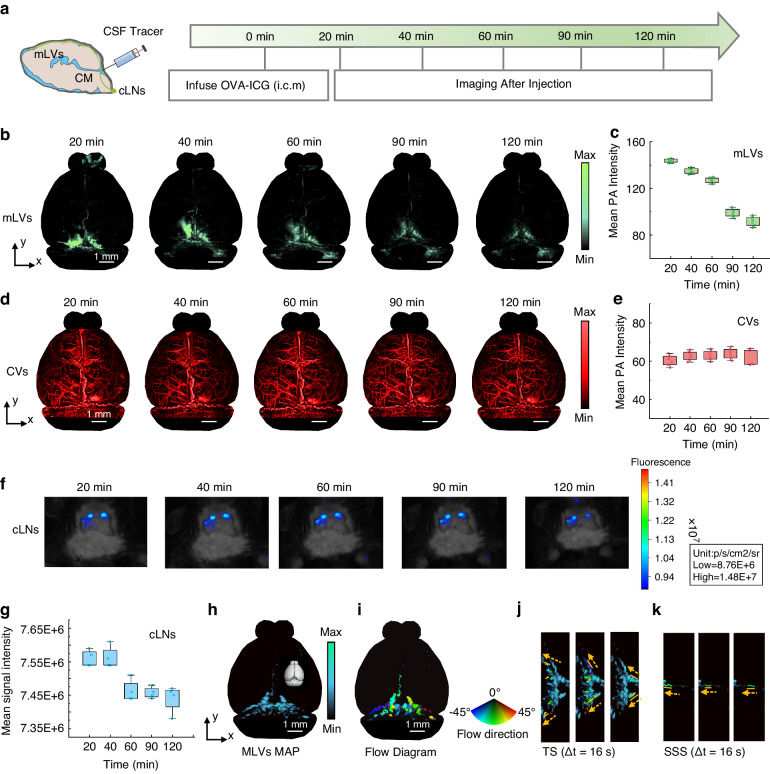
PA insights on drainage and clearance of mLVs. **a** The imaging process that the mouse is subjected to imaging after tracer injection (*n* = 3). **b** MAP images of mLVs at 20, 40, 60, 90, and 120 min after injection. **c** Quantitative analysis of mean PA intensity of mLVs. **d** MAP images of CVs at 20, 40, 60, 90, and 120 min after injection. **e** Quantification analysis of mean PA intensity of CVs. **f** In vivo fluorescence images of cLNs after injection. **g** Fluorescence intensity of cLNs. **h** PA imaging of the mouse cortex after injection of OVA-ICG. **i** The flow direction of the tracer. **j** and **k** The tracer flow over time, where orange arrows show the direction of their flow, and the detailed information of flow direction at TS and SSS regions are located, respectively

To study the functional characterization of mLVs, PA images of mLVs and CVs are acquired at different time points (Fig. 5a and Table S3). The images are taken at 20, 40, 60, 90, and 120 min after injection. The PA data of mLVs are processed to generate maximum amplitude projection (MAP) images in the horizontal views at different time points (Fig. 5b). A noticeable difference is observed in the overall view of mLVs at different time points, with fewer signals detected in later time points compared to earlier ones. Following tracer injection, the mean PA intensity significantly increased, showed high fluctuations at 20 minutes, and then gradually decreased thereafter (Fig. 5c and Fig. S13), which indicated the drainage and clearance of mLVs.

The PA images of CVs are minimally affected by the injection period (Fig. 5d). Putting into the same perspective where extracting the mean PA intensity at each time point observes relatively stable values of CVs (Fig. 5e and Table S3). Additionally, no significant difference in cerebral blood flow is observed between different time points (Fig. S14). The steady flow properties of hemoglobin emphasize the drainage and clearance characteristics of the tracer through mLVs.

Further insights into the process are obtained by analyzing the fluorescence intensity of cLNs (Fig. 5f and Table S3). Concretely, the fluctuation in cLNs intensity (Fig. 5g) mirrored those observed in mLVs of PA images, with a peak period of cLNs drainage occurring around 20–40 min after CM injection, followed by a decrease. This means that the tracers are drained from CSF into cLNs through mLVs.

Moreover, our system monitors tracer migration in vivo over short distances (< 200 μm) and estimates flow direction (See Supplementary Text and Fig. S15). It should be noted that the injected OVA-ICG can exist in mLVs as both single molecules and OVA-ICG clusters, contributing to the strong signals observed in the images. These signals are clearly visualized in transverse sections (Fig. 5h and Movie. S2). A motion-contrast image is created from B-scan images with an interval time of 0.8 s and overlaid on the mLVs image (Fig. 5i). The colors of the arc disk represent the flow direction of OVA-ICG, ranging from –38° to 66°. Detailed movements of the tracer are visualized, with the flow direction indicated by orange arrows. The flow direction of the tracer at TS regions (Fig. 5j) and SSS regions (Fig. 5k) aligned with previous findings^5,22^. These results demonstrate that this system aids in understanding the dynamics of drainage and clearance through mLVs, providing a pathway for subsequent research on brain diseases, such as neurodegenerative diseases.

### PA insights on mLVs function in Alzheimer’s disease and young mice

**Fig. 6:**
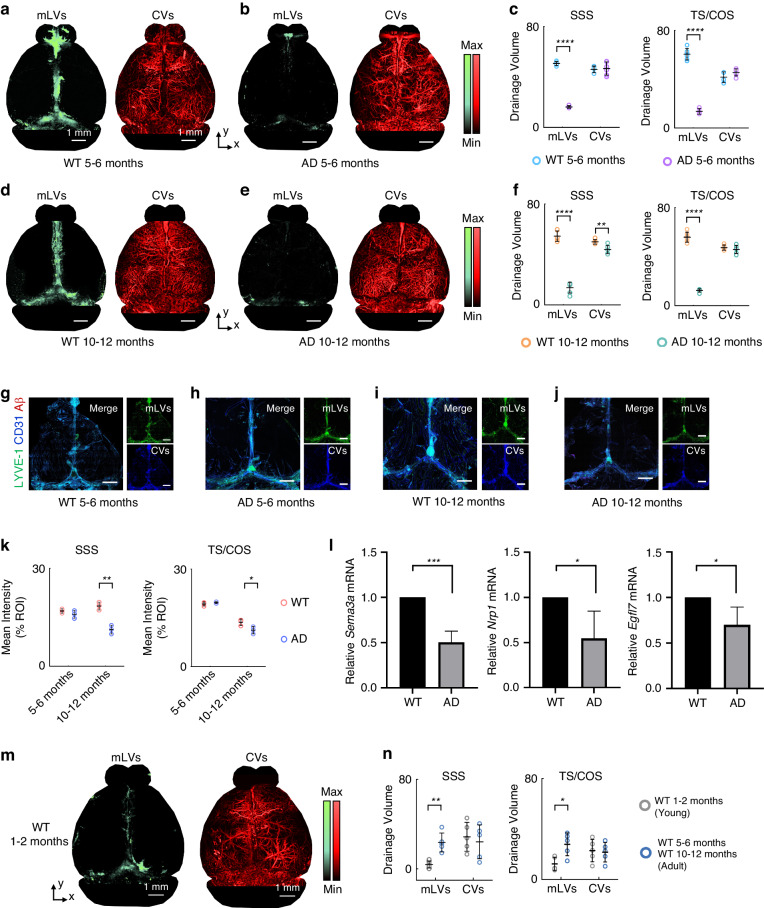
PA insights on mLVs function in Alzheimer’s disease and young mice. **a**, **b** Representative MAP PA image of mLVs and CVs from WT 5–6 months, AD 5–6 months. **c** Graphs showing the drainage volume by mLVs and CVs between WT 5–6 months and AD 5–6 months mice. **d**, **e** Representative MAP PA image of mLVs and CVs from WT 10–12 months, AD 10–12 months. **f** Graphs showing the drainage volume by mLVs and CVs between WT 10–12 months and AD 10–12 months mice. **g**–**j** Representative images of meningeal whole mounts (WT 5–6 months), AD 5–6 months, WT 10–12 months, and AD 10–12 months, stained for LYVE-1 (green), CD31 (blue), and Aβ (red), scale bar: 1 mm. **k** Graphs showing mean intensity by mLVs between 5–6 months and 10–12 months mice. **l** Relative mRNA levels of *Sema3a*, *Nrp1* and *Egfl7* in the meninges of mice. **m** Representative MAP PA image of mLVs and CVs from WT 1–2 months. **n** Graphs showing the drainage volume by mLVs and CVs between young mice (WT 1–2 months) and adult mice (WT 5–6 months and WT 10–12 months). Results in (**c**) and (**f**) are presented as mean ± s.e.m.; *n* = 5 per group; two-tailed unpaired Student’s *t* test. Detailed statistical information is shown in Table S4 and S5

The discovery and characterization of mLVs have prompted an assessment of their role in waste clearance from the CNS. Adult (5–6 months and 10–12 months) 5xFAD transgenic mice in PA modality all exhibit obvious meningeal lymphatic dysfunction (Fig. 6a–f). The drainage volume of CVs in AD mice (5xFAD transgenic mice) is comparable to that in the wild-type (WT) mice at 5–6 months, but the drainage volume of mLVs at 5–6 months carried an obvious drainage volume decrease and only ~32% that of WT mice (SSS: 16.26 ± 1.02 and 50.73 ± 1.56%, respectively; TS/COS: 13.70 ± 2.33 and 60.57 ± 4.71%, respectively) (Fig. 6c and Table S4). In addition, the drainage volume of CVs in the SSS regions in AD mice aged 10–12 months is slightly lower than that in WT mice (43.96 ± 3.31 and 50.05 ± 1.88%, respectively). While the drainage volume of mLVs at 10–12 months is only ~25% that of WT mice (SSS: 13.76 ± 4.31 and 54.54 ± 4.14%, respectively; TS/COS: 12.41 ± 1.30 and 55.88 ± 4.14%, respectively) (Fig. 6f and Table S5). This demonstrates the continued decline in mLVs drainage as Alzheimer’s disease progresses. As a control, we imaged and analyzed the mLVs and CVs structure of the above mice using the mLVs marker Lyve-1 in combination with vascular marker CD31 in vitro (Fig. 6g–j). Imaging of whole-mount meninges revealed the structural lack of mLVs in AD mice aged 10–12 months (SSS: 11.26 ± 1.34 and 18.45 ± 1.22% ROI, respectively; TS/COS: 11.20 ± 1.13 and 13.63 ± 0.99% ROI, respectively), whereas aged 5–6 months, this loss is not evident (SSS: 15.91 ± 1.20 and 16.92 ± 0.66% ROI, respectively; TS/COS: 19.72 ± 0.25 and 19.27 ± 0.62% ROI, respectively) (Fig. 6k and Table S6). In contrast, there is a significant decrease in lymphatic vessel coverage along the SSS, TS, and the confluence of sinuses (COS) in the PA image of AD mice aged both 5–6 months and 10–12 months. The functional impairment of mLVs imaged DCF-PAM is more evident than in images of fluorescently labeled mLVs from the same mice. Next, we verified the cLNs kinetic biodistribution of OVA-ICG administration between WT mice and AD mice. 20-40 minutes after administration of OVA-ICG, the intensities of OVA-ICG fluorescent signals in cervical regions (Fig. S16) are still significantly weaker in AD mice than in WT mice. Interestingly, The PA image of mLVs has fewer drainage volume aged 10–12 months observed compared to 5–6 months (13.76 ± 4.31 and 16.26 ± 1.02%, respectively). These results suggested that impaired drainage of mLVs may be due to Aβ deposition or aging.

It is worth noting that AD mice in IF imaging do not exhibit an obvious reduced structure of mLVs, but the deterioration of the lymphatic function at the meninges is clearly observed in PA imaging in vivo. To verify the impairment in the mLVs function of AD mice, we detected the expression of genes relative to lymphatic vessel structure and function in mLVs of AD mice. Although the gene transcripts *Foxc2*, *Celsr1*, *Pkd1*, and *Fat4*, which lymphatic valve development and recruitment of smooth muscle cells, are not significantly changed^24^, mRNA levels of the transcription factors *Sema3a*, *Egfl7*, and *Nrp1*, which regulate cell polarization and vascular development, are down-regulated (Fig. 6l and Fig. S17).

Finally, to explore the cause of mLVs function impairment in AD mice, we hypothesized that the impairment in the mLVs function may be controlled by Aβ deposition. Fluorescently labeled Aβ antibodies throughout the meninges are observed, accompanied by an increase in Aβ deposition at 10–12 months in AD mice (Fig. 6j). Therefore, the impairment in the mLVs function may emerge with Aβ deposition in these mice. Another, adult mice aged 5–6 months and 10–12 months demonstrate increased brain perfusion by CSF macromolecules compared to young counterparts aged 1–2 months (Fig. 6m). Enhanced brain perfusion by CSF in adult mice is accompanied by an extension in mLVs density (Fig. 6n and Table S7). The reduced drainage volume of mLVs in young mice compared to adult mice is likely because of incomplete development of the meningeal function. Collectively, these results indicate that the mLVs function in the pathogenesis stage of AD mice has been disrupted, as well as apparent mLVs are abundant in adult mice compared to young mice. These results indicated that DCF-PAM images efficiently to mLVs and can serve as a good application direction for mLVs functional imaging.

## Discussion

The strategy that we have described here includes every step after probe syntheses to final imaging rendering, along with the extract of the various quantitative analyses. DCF-PAM is utilized to achieve wide-field intravital imaging of mLVs. From the images generated, we can distinguish between mLVs on the meninges with glymphatic pathways within the brain parenchyma, and quantify the influence of CSF drainage and clearance. The availability of the system should be seen as an incentive for mLVs imaging of in vivo applications. The approach can be used to try probes injected into CM without euthanizing the mouse and dissecting the meninges. We believe that the approach presented here could be used whenever a brain disease related to the mLVs is observed in vivo. To be gratified, we have successfully characterized the perivascular cerebrospinal fluid dynamics of the glymphatic pathway system^40^. Therefore, it is exhilarating to realize the high-resolution imaging in vivo of mLVs using DCF-PAM.

Photoacoustic microscopy is the high-resolution imaging modality that has been achieved for significant applications in some biomedical fields^41–43^. In the last decades, the distinct signatures of the photoacoustic microscopy enabled the quantification of more comprehensive information such as angiogenesis^44^, oxygen saturation (sO2)^45^, oxygen extraction fraction, and cerebral blood volume^46,47^, and also enhanced the ability of the hybrid system to recover dense vascular networks^48–50^ and hemodynamic and morphological variations in both superficial and deep tissues^51–54^. Furthermore, various photoacoustic system has been explored in brain metabolism^32,55^, brain injury^56–59^, state connectivity^60^, and brain response^61,62^ by employing different laser illumination and detection schemes. These technologies have been achieved in the study of in vivo high-resolution imaging of the brain, whereas they almost concentrate on CVs rather than mLVs. Of course, the imaging of mLVs should also be studied. Partial studies have been made in some pure-optical ways, IF way, as well as MRI way, but it is hard to visualize the structure and function of mLVs with high resolution in vivo. DCF-PAM allows simultaneous visualization of CVs and mLVs with high resolution in vivo. Vascular organizations are spread throughout the whole view due to the significant signal of hemoglobin, and mLVs are typically characterized by concentrated near the TS and SSS. The images of CVs and mLVs feature different experimental characteristics, reflecting the differences in their organizations. We hope that with the system provided in this paper, accurate characterization of CVs and mLVs in vivo will be feasible. DCF-PAM enables stereoscopic images of CVs and mLVs in vivo. Through the self-developed hollow ultrasound transducer^63,64^ and light-acoustic confocal system design, the obtained stereoscopic images can maintain a good signal-to-noise ratio and resolution within a depth range of 3.75 mm.

Using mLVs images analyzed, we notice that the probes are especially important in the imaging process of mLVs. The OVA-ICG as a tracer used is a macromolecule in its form. This means that probes come from CSF by drainage and clearance, and not enough covered entire mLVs to perform a detailed visualization of mLVs. All illuminated signal points tend to only show the trajectories of the probe in all mLVs. As such, labeled probes should be used to label the entire network of mLVs in the future. One can note that, even though the mouse needs to euthanize in the IF imaging, the conjugates of antibody and fluorescent dye are performed as labeled probes, and that should be taken into account when synthesizing PA probes. If labeled PA probes will be synthesized and injected into CM, the final reconstructed images may reveal entire mLVs.

For the DCF-PAM, the device based on the two-dimension motorized translation stage has a wide-view scanning range. This makes the image obtained to cover the whole brain of a mouse. In vivo, wide-view monitoring seems to include more structured information about the sample, whereas CVs and mLVs have many real-time features of flow behavior. The duration of one PA imaging in the brain of DCF-PAM does not allow the real-time representation of CVs and mLVs as it needs long acquisition times. The scanning speed is critical to the real-time recording of a series of dynamic changes. If the system in the strategy is able to use some high-speed scanning device, such as a 2-axis fast galvanometer scanner, providing high-frame-rate scanned images and realizing real-time whole-brain recording results will be possible. Our previous study has a dual-raster-scanned PA microscope that integrates a two-dimensional motorized translation stage for large field-of-view imaging and a 2-axis fast galvanometer scanner for real-time imaging^65^. In the future, we will have chosen this system to update the dual-wavelength system. The idea appears much better than those of many other methods. It will improve the applicability of the strategy by allowing wide-view monitoring and real-time recording.

In summary, we have proposed an approach for high-resolution image CVs and mLVs in vivo using DCF-PAM. When the advent of relative brain diseases or injury, the system devised may also help to compare and characterize such diagnoses and treatment of diseases. The use of the system is seen potential to increase new imaging technology and method and realize better and faster in vivo imaging of CVs and mLVs. It will facilitate brain study that relevant aspects of the nervous system and immune system by researchers, and allow them to take advantage of the existing condition and develop new application directions.

## Materials and methods

### Animal subjects

Young (1–2 months old) and adult (5–6 months, 10–12 months old) male C57BL/6 J WT mice are purchased from Guangdong Medical Laboratory Animal Center. The transgenic mice (5xFAD) are provided by Sun Yat-sen Memorial Hospital, Sun Yat-sen University. All experimental mice are of a C57BL/6 background, and WT and AD mice are paired from the litters and housed under the same living conditions. In-housed bred male transgene carriers and non-carrier (WT) littermates are used at different ages. Mice are housed in a temperature and humidity-controlled room, maintained in a 12 h/12 h light/dark cycle. All mice are fed with regular rodent chow and sterilized tap water ad libitum. All animal studies were reviewed and approved by the Institutional Animal Care and Use Committee of Hainan University (approval number: HNUAUCC-2021-00112).

### Cisterna magna Injection

Mice are anaesthetized by inhaling 2% isoflurane air. The hair of the neck is shaved and cleaned with iodine and 70% ethanol, an ophthalmic solution is placed on the eyes to prevent drying, and the head of the mouse is secured in a stereotaxic frame. After making a skin incision, the muscle layers are retracted, and the CM is exposed. Using a Hamilton syringe (coupled to a 30-gauge needle), the volume of desired tracer solution is injected into the CSF-filled cisterna magna compartment at a rate of about 2.5 μl per minute^66^. After injecting, the syringe is left in place for at least 2 min to prevent backflow of CSF. The neck skin is then sutured. The method of intra-cisterna magna injection is used to administer 10 μl of OVA-ICG. The tracers are imaged at the indicated time points and analyzed.

### Photoacoustic Imaging

For PA imaging of the mouse brain, the hair of the normal mouse is first removed using a shaver and depilatory cream. Then, the mouse is removed scalp. The skull is kept intact. Imaging is performed with the scanning range of 12 × 12 × 3.75 mm^3^, and fast-axis scanning speed of 10 mm s^–1^, and the scanning time is about 12 min. During scanning, to avoid motion artifacts in living mice, firstly, the degree of anesthesia of the mice is controlled through gas anesthesia, and analgesia and central muscle relaxation medication are administered to reduce the impact of breathing. Secondly, during the process of acquiring images, the bandpass filter is used to smooth the PA signal, and median filtering is performed on every 4 A-line data to eliminate the noise. Finally, after acquiring the 3D PA data, the motion correction algorithm and bilinear interpolation are applied to further reduce motion artifacts in the image^67^.

In the phantom experiments, the 30-gauge needle is inserted into the mouse brain in vitro. To measure the penetration depths, 2500 B-scan images are acquired, and each B-scan image contained 500 A-lines. To evaluate the lateral resolution, the edge spread function as well as the corresponding line spread function are calculated perpendicular to the blade edge. To evaluate the axial resolution, the acquired A-line signals are performed Hilbert transformation and measured the full-width-at-half-maximum. 2500 B-scans to cover the FOV are acquired as a set of coronal views, and MAP to each B-scan image is carried out so that there were 2500× 1000 pixels in total in one image of the horizontal view. In sequential imaging experiments, PA images are continuously acquired for approximately 2 hours post-injection.

### MRI acquisitions

All MRI acquisitions are performed in a 9.4 T BioSPEC system (Bruker, Germany). The mouse is placed in the MRI apparatus and maintained under light anesthesia with isoflurane (1–1.25% in oxygen). Before and after injecting DTPA-Gd into the CSF, a series of post-contrast T1-weighted images are taken through the head with the following parameters: TR = 500 ms, TE = 6 ms, FOV = 20× 20 mm^2^, slice thickness = 0.7 mm, number of slices = 17 and NEX = 2. The total acquisition time is about 147 s.

### Immunohistochemistry and Imaging

Mice are euthanized by intraperitoneal (i.p.) injection with an overdose of anesthesia (20 mg kg^–1^ pentobarbital sodium) and transcardially perfused with PBS. The skin is removed from the head and the muscle is stripped from the bone. Then, the mandibles and skull rostral to maxillae are removed. Whole-mount skullcap is fixed while still attached to the skull cap in 4% paraformaldehyde for 12 h at room temperature. The meninges are then dissected from the skullcap. The tissue is rinsed in PBS and 0.5% Triton X-100 containing 5% bovine serum albumin for 1 h at room temperature. This blocking step is followed by incubation with appropriate dilutions of primary antibodies: anti-LYVE-1-Alexa Fluor 488 (eBioscience, 1:200), anti-CD31 (Millipore, MAB1398Z, 1:200) in PBS, and 0.5% Triton X-100 overnight at 4 °C. Meningeal whole-mounts are then washed three times for 10 min at room temperature in PBS and 0.5% Triton X-100 followed by incubation with appropriate Alexa Fluor 555 (Thermo Fisher Scientific, 1:400) in PBS and 0.5% Triton X-100 overnight at 4°C. Alternatively, incubate overnight at 4°C with the appropriate Anti-β-amyloid, 1-16 Antibody (Biolegend, 1:400), Alexa Fluor 647 (Thermo Fisher Scientific, 1:400). After incubating, the tissue is washed three times for 10 min with PBS and 0.5% Triton X-100 at room temperature and mounted with an IF sealing agent and glass coverslips^68^. Preparations are stored at 4°C for no more than one week until images are acquired using a confocal microscope (LSM 880 Confocal Laser Scanning Microscope, Zeiss).

### Laser speckle

Mice are anesthetized by isoflurane. An incision is done along the midline to separate the skin of the skull and laser speckle is used to detect mice’s cerebral blood flow. Laser speckle blood flow images are recorded and used to identify the regions of interest. Within these regions, the mean blood flow index is calculated in real time.

### Quantitative real-time PCR

Relative mRNA expression levels of target genes are measured by qPCR. Total RNA is extracted from cells or tissues with RNAiso Plus (TaKaRa, D9108A) following the manufacturer’s instructions, and cDNA is generated using a ReverTra Ace qPCR (Quantitative Real-time PCR) RT Kit (TOYOBO, FSQ-301). Real-time PCR is performed using SYBR Green PCR mix on a CFX ConnectTM Real-Time System normalized against GAPDH. The 2^-ΔΔCt^ method is used to calculate relative gene expression levels. *Gapdh* is amplified as an internal control. Sequences of all primers for amplification of genes used in experiments are listed in Table S8.

### Statistical analysis

After acquiring and storing the origin PA data, the median filtering algorithm and wavelet filtering algorithm are implemented based on a user-defined program for LabVIEW software. For each PA matrix, the various planes of the PA matrix are extracted. The horizontal plane and coronal plane of PA images are obtained. By applying for interpolated algorithm and reconstruction program, it is able to compute the MAP and depth-encoded by processing them through MATLAB program. The 3D image could be generated by arranging each matrix according to the time vector.

The MR images and the IF images are rendered with the software tool, RadiAnt, and ZEN, respectively. The merged images combing all the point-to-point positions of each pixel in a maximum-intensity projection fashion are fused using the software ImageJ, containing PA/PA image and IF/IF image. The ratio between the CVs signal area and the mLVs signal area is accessed to compare image features based on the different imaging methods. The mean PA intensity is calculated to reflect the condition of drainage and clearance. The mean PA intensity of a specific region is calculated, and the total value of the target region divided by the area of the target area can represent the average amount of tracer in the target region.

We estimated the local motion vectors from the consecutive frame and plotted their direction with color. According to the previous study, our system would capture the direction of lymph flow because the B-scan scanning velocity is less than the lymph velocity^69–72^. The mLVs that contained flowing tracers are first manually identified from the consecutive B-scan images. Run traces at points along these signals are then extracted. After thresholding the set of maximum amplitude, the images are fitted to estimate a flow direction.

Statistical analysis is conducted in SPSS software and GraphPad Prism 8. Data are expressed as the mean ± SEM. Significant differences are compared as noted in figure legends, using Student’s *t* test for statistical analysis in two-group comparison, and the differences are considered statistically significant at *p* < 0.05. Specific statistical parameters are detailed in the figure legends.

### Supplementary information

Supplementary Information for: Ultra-high spatio-temporal resolution imaging with parallel acquisition-readout structured illumination microscopy (PAR-SIM)

The stereoscopic PA imaging of mLVs, glymphatic pathways, and cerebral vessel

The flow direction of the tracer

## Data Availability

The data that support the findings of this study are available from the corresponding author upon request.
