# Supplementary material for: Advancing insights into in vivo meningeal lymphatic vessels with stereoscopic wide-field photoacoustic microscopy
Source: Light Sci Appl. 2024 Apr 25;13:96. doi: 10.1038/s41377-024-01450-0 (PMC11045809; doi:10.1038/s41377-024-01450-0)
Supplement: Supplementary file 1 — Supplementary Information for: Ultra-high spatio-temporal resolution imaging with parallel acquisition-readout structured illumination microscopy (PAR-SIM) [file 41377_2024_1450_MOESM1_ESM.docx]

**Supplementary Information for**

Advancing insights into *in vivo* meningeal lymphatic vessels with stereoscopic wide-field photoacoustic microscopy

Fei Yang^1,2^, Zhiyang Wang^1,2^, Wenbin Shi^1,2^, Miao Wang^3^, Rui Ma^1,2^, Wuyu Zhang^1,2^, Xipeng Li^1,2^, Erqi Wang^1,2^, Wenjie Xie^1,2^, Zhan Zhang^4,5^, Qi Shen^1,2,*^, Feifan Zhou^3,*^, Sihua Yang^1,2,6,*^

*e-mail: shenqi@scnu.edu.cn; [zhouff@hainanu.edu.cn](mailto:zhouff@hainanu.edu.cn); [yangsh@scnu.edu.cn](mailto:yangsh@scnu.edu.cn).

^1^MOE Key Laboratory of Laser Life Science & Institute of Laser Life Science, South China Normal University, Guangzhou 510631, China

^2^Guangdong Provincial Key Laboratory of Laser Life Science, College of Biophotonics, South China Normal University, Guangzhou 510631, China

^3^Key Laboratory of Biomedical Engineering of Hainan Province, School of Biomedical Engineering, Hainan University, Haikou 570100, China

^4^Department of Neurology, Sun Yat-sen Memorial Hospital, Sun Yat-sen University, Guangzhou 510120, China.

^5^Guangdong Province Key Laboratory of Brain Function and Disease, Zhongshan School of Medicine, Sun Yat-sen University, Guangzhou 510120, China.

^6^Guangdong Basic Research Center of Excellence for Structure and Fundamental Interactions of Matter, South China Normal University, Guangzhou 510006, China

These authors contributed equally: Fei Yang, Zhiyang Wang.

Supplementary Text

**CuS synthesis**

An aqueous solution was first prepared by mixing 20 mL of water, 0.4 mL of PVP (polyvinylpyrrolidone); MW = 30 000, Beijing Chemical Co.) solution (1.0 wt %), and 0.2 mL of CuBr_2_ solution (0.1 M). Then 1.0 mL of ascorbic acid solution (0.1 M) was added to the solution, and the mixture was stirred for 3 min and became turbid shortly, indicating the formation of CuBr particles. Then, 0.4 mL of Na_2_S solution (0.1 M) was added to the turbid precursor solution under stirring, which was then kept for 30 min at room temperature without stirring, resulting in the formation of poorly crystallized Cu_2_S hollow spheres. This brown reaction mixture was maintained at 160 °C for 24 h, leading to the formation of well-crystallized CuS hollow spheres. The resultant CuS products are collected by centrifugation, washed with water several times, and dried in air ^73^.

**The spatial resolution of DCF-PAM using CuS**

In order to characterize the optical sensitivity of the prepared CuS, an ultraviolet-visible (UV-vis) spectrophotometer is used to measure the UV-vis absorbance spectra of CuS (Fig. S1a). The size of the synthesized CuS is verified by dynamic light scattering with an average diameter of 700 nm (Fig. S1b). These results demonstrate that the prepared CuS has good performance and satisfies the system's requirement of characterization. PA images of CuS with 780 nm are selected with various regions. The Gaussian-fitted full-width-at-half-maximum (FWHM) of the nanoparticle in region #1 is 11.36 μm. For region #2: 10.64 μm. For region #3: 8.96 μm. For region #4: 7.63 μm. For region #5: 8.53 μm. For region #6: 9.43 μm. For region #7: 10.33 μm. For region #8: 10.43 μm. For region #9: 11.78 μm. For region #10: 23.8 μm (Fig. S2). PA images of CuS with 532 nm are selected with various regions. The Gaussian-fitted full-width-at-half-maximum (FWHM) of the nanoparticle in region #1 is 14.8 μm. For region #2: 14.5 μm. For region #3: 10.03 μm. For region #4: 5.69 μm. For region #5: 7.25 μm. For region #6: 9.34 μm. For region #7: 11.37 μm. For region #8: 15.59 μm. For region #9: 16.48 μm. For region #10: 18.05 μm (Fig. S3). By the curve of the signal to noise ratio (SNR) along the depth direction, the system approach exhibits excellent contrast and spatial resolution (Fig. S4).

**OVA-ICG toxicity evaluation**

To better demonstrate the low-damage properties of OVA-ICG, we conducted a series of toxicity assessment experiments. Firstly, we incubated different concentrations of OVA-ICG and clinically used concentrations of ICG with meningeal lymphatic endothelial cells for 24 hours; we then quantitatively evaluated cell viability through the CCK8 kit, as shown in Fig. S7. Experimental results prove no obvious toxicity. Then, OVA-ICG is injected into the cisterna magna and then sacrificed after 48 hours, where hematological parameters are investigated systematically (Fig. S8). Compared with the control group, no obvious abnormal changes are observed for the OVA-ICG group, indicating that these tracers did not cause obvious toxicity in the mice.

**The assessment of flow direction**

The assessment of flow direction is divided into the following main steps (Fig. S15): (1) a sequence of PA volume data is quickly obtained, (2) track the position changes of PA signals in consecutive B-scan images, (3) calculate the maximum amplitude in a sequence of B-scan images to obtain the maximum projection on the X-Y plane, (4) to monitor tracer migration over short distances (< 200 μm) *in vivo*, B-scan images every 20 frames are divided into the start frame and the end frame of the flow direction, (5) in the maximum projection image, take the coordinates of the center pixel of the photoacoustic signal to calculate △x and △y of the start frame and the end frame, and the movement angle = arctan (△x/△y), (6) the calculated angle to each maximum projection position of the 20 frames is assigned, and finally the calculated results of the divided areas are spliced to obtain a flow diagram of the whole brain.


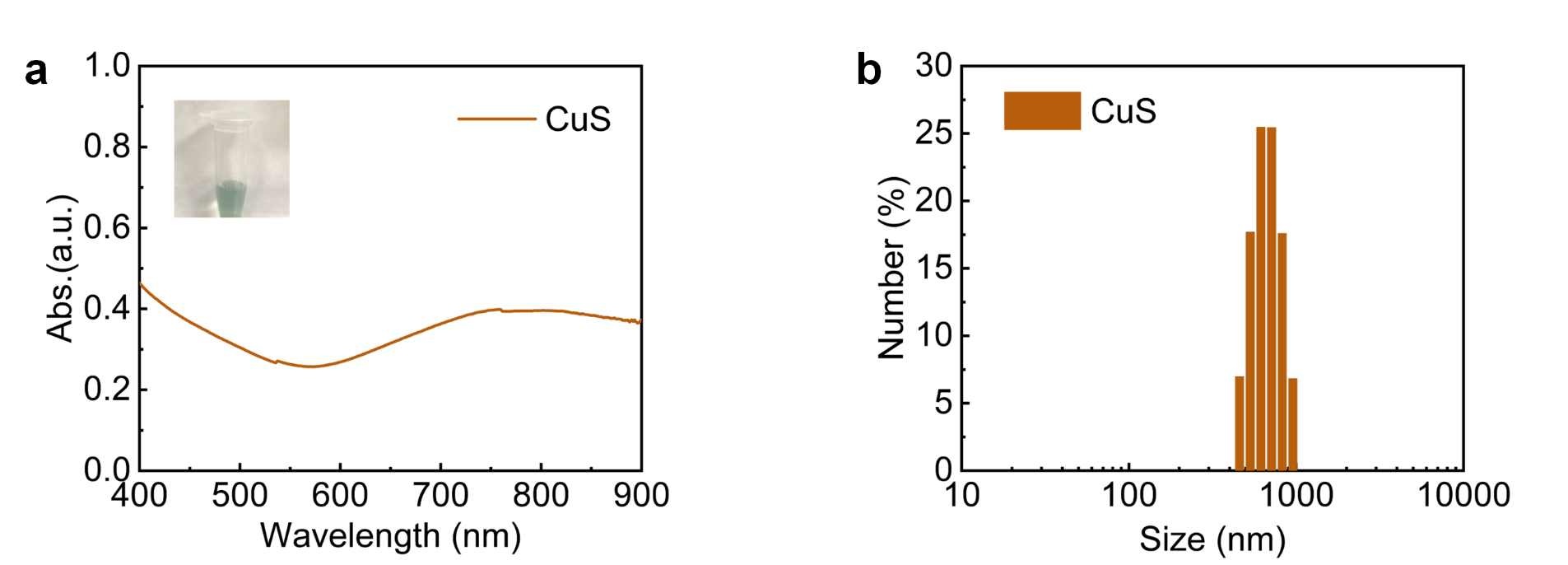


Fig. S1.

**Characterization of CuS for imaging at spatial resolution.** **a** Obvious absorbance of broad spectra of CuS. **b** The size of the synthesized CuS.


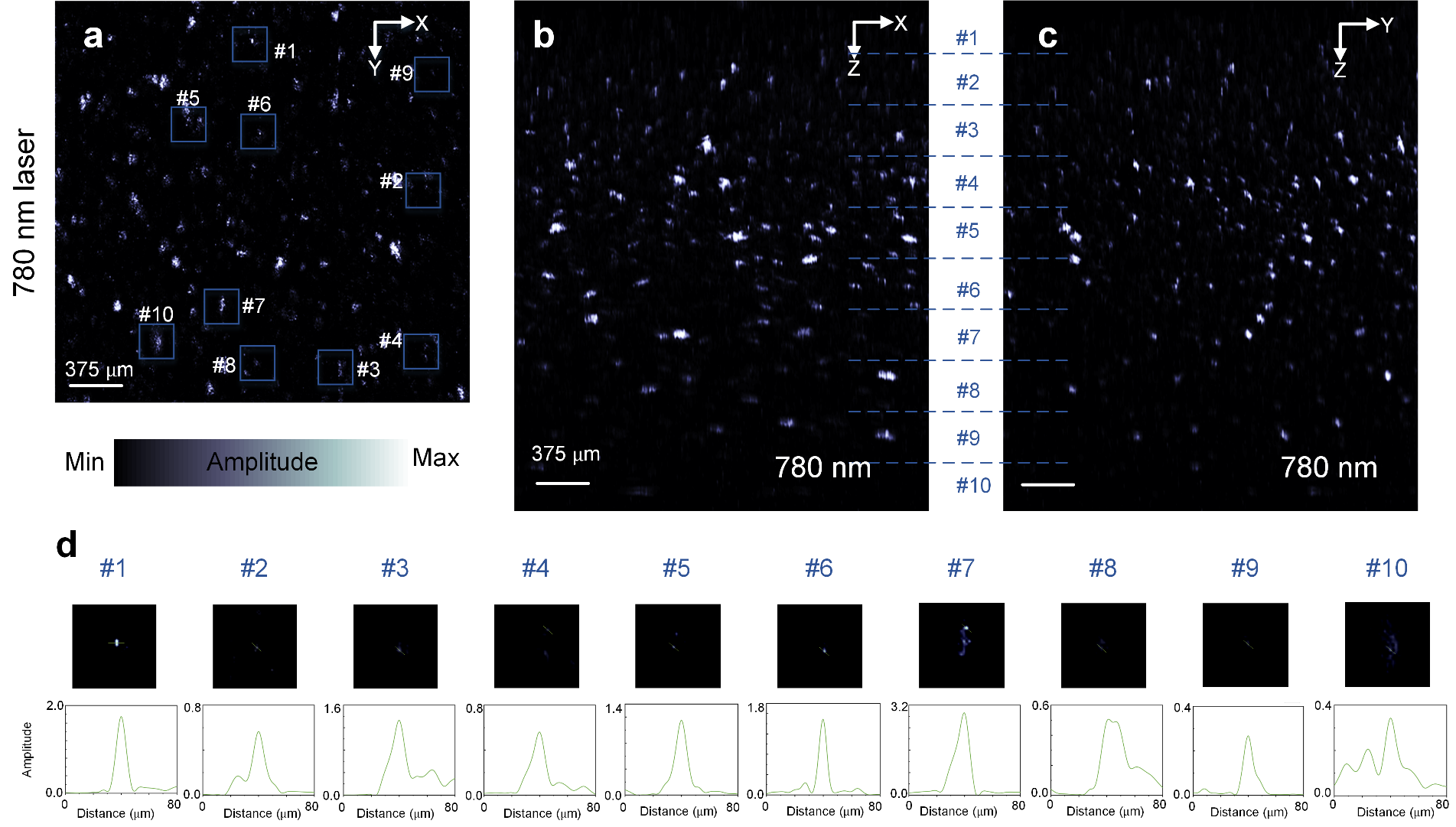


Fig. S2.

**PA images of CuS with 780 nm laser.** **a** PA image of CuS with 780 nm laser at XY direction. **b** PA image of CuS with 780 nm laser at XZ direction. **c** PA image of CuS with 780 nm laser at YZ direction. **d** Magnified views of the selected images in the blue regions together with their signal profiles along the corresponding green lines.


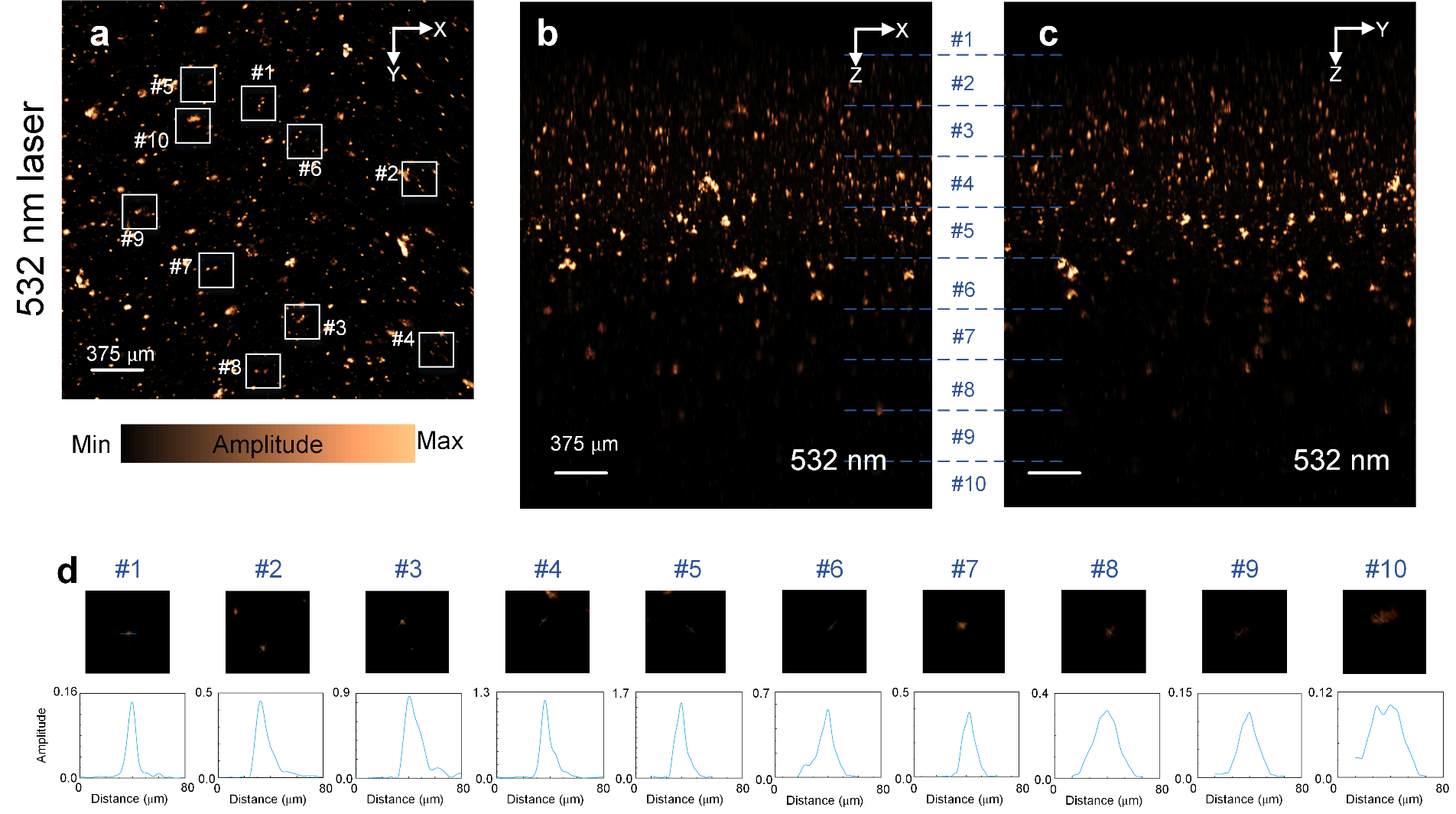


Fig. S3.

**PA image of CuS with 532 nm laser**. **a** PA image of CuS with 532 nm laser at XY direction. **b** PA image of CuS with 532 nm laser at XZ direction. **c** PA image of CuS with 532 nm laser at YZ direction. **d** Magnified views of the selected images in the white regions together with their signal profiles along the corresponding blue lines.


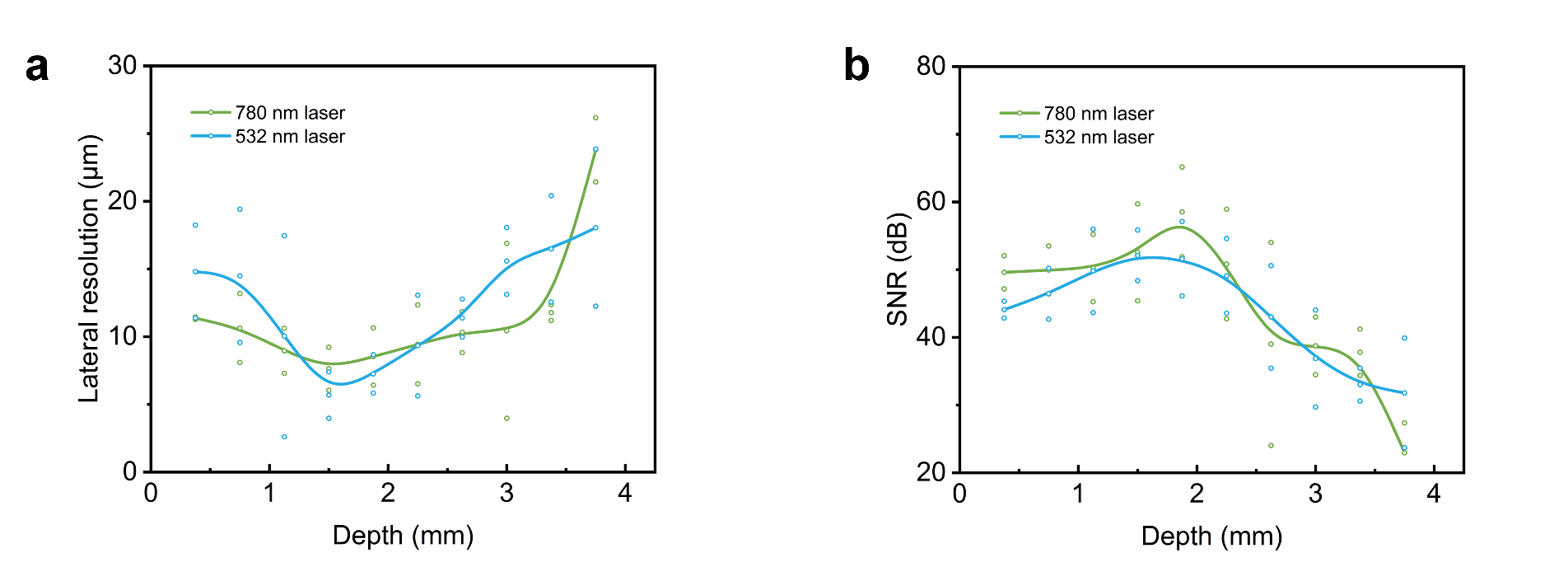


Fig. S4.

**The contrast and spatial resolution along the depth direction.** **a** The curve of the lateral resolution along the depth direction. **b** The curve of the Signal to Noise Ratio (SNR) along the depth direction.


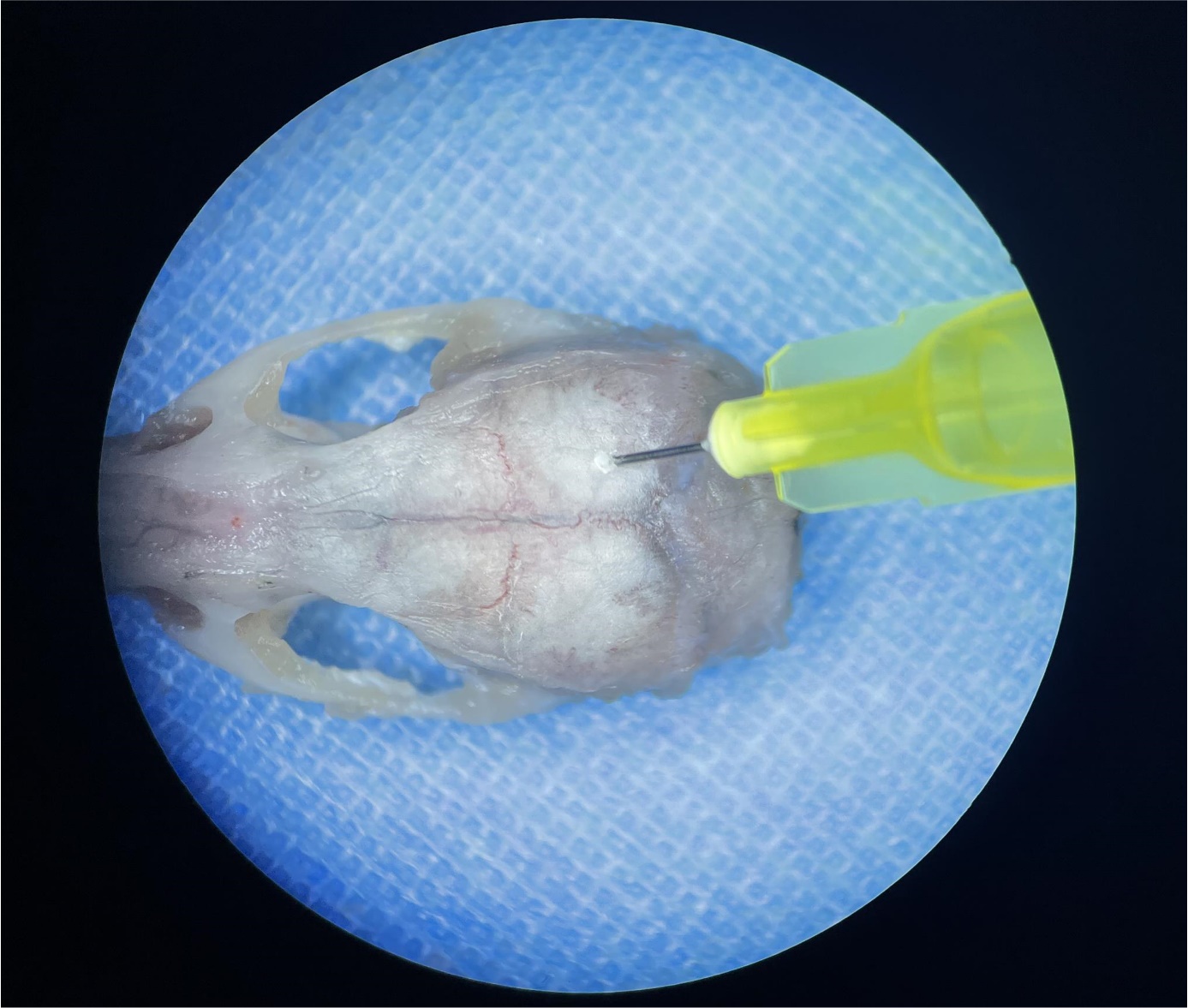


Fig. S5.

**The penetration depth through the intact skull was measured.** A real environment with a 30-gauge needle inserted into the mouse brain is simulated.


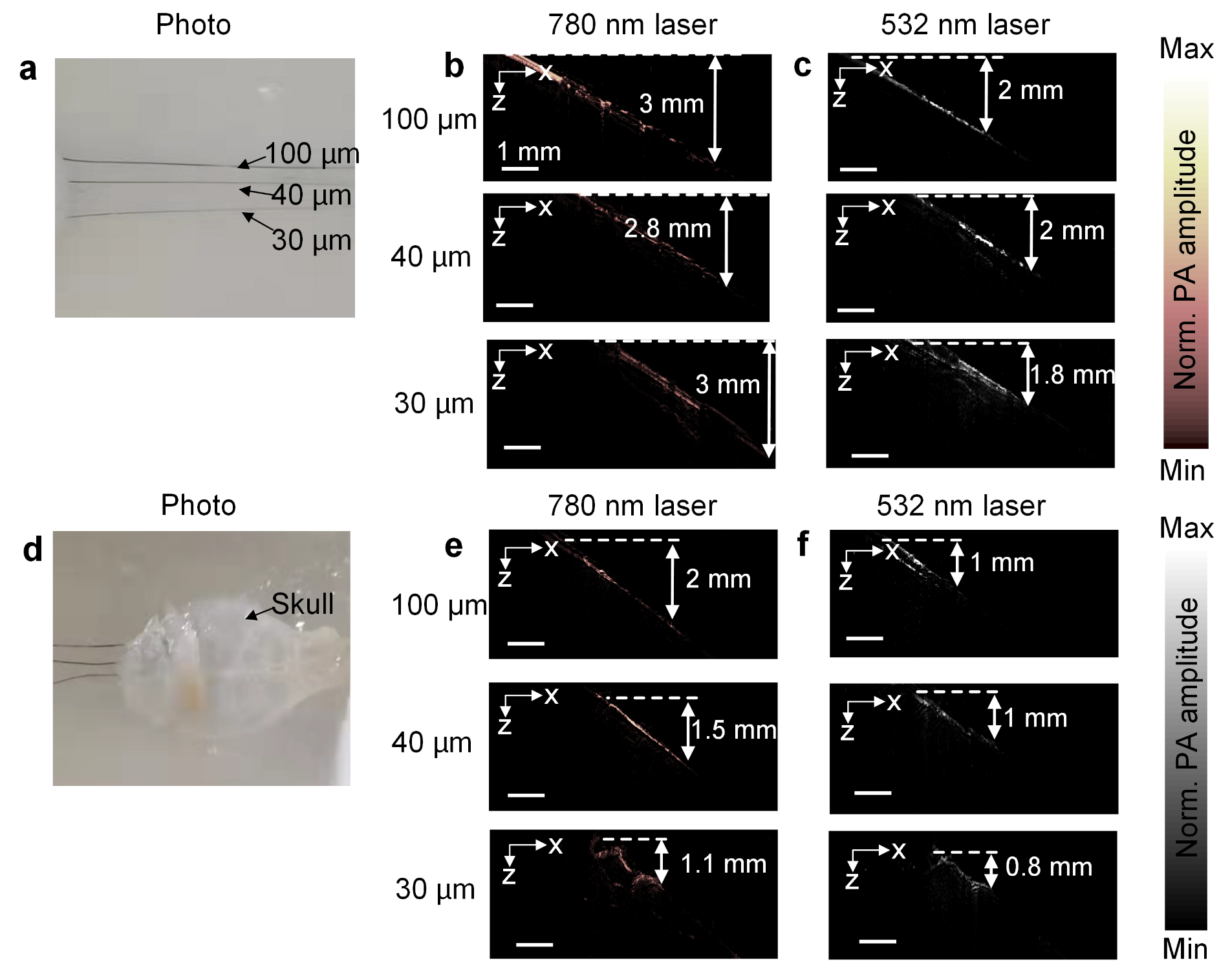


Fig. S6.

**Characterization of the imaging depth. a** The imaging depths for two wavelengths are measured by obliquely inserting tungsten wire into the agar. **b** The imaging depths are 3 mm, 2.8 mm, and 3 mm using a 780 nm wavelength with the size of 100 μm, 40 μm, and 30 μm. **c** The imaging depths are 2 mm, 2 mm, and 1.8 mm using a 532 nm wavelength with the size of 100 μm, 40 μm, and 30 μm. **d** The imaging depths for two wavelengths are measured by obliquely inserting tungsten wire into agar under the skull. **e** The imaging depths are 2 mm, 1.5 mm, and 1.1 mm using a 780 nm wavelength with the size of 100 μm, 40 μm, and 30 μm. **f** The imaging depths are 1 mm, 1 mm, and 0.8 mm using a 532 nm wavelength with the size of 100 μm, 40 μm, and 30 μm.


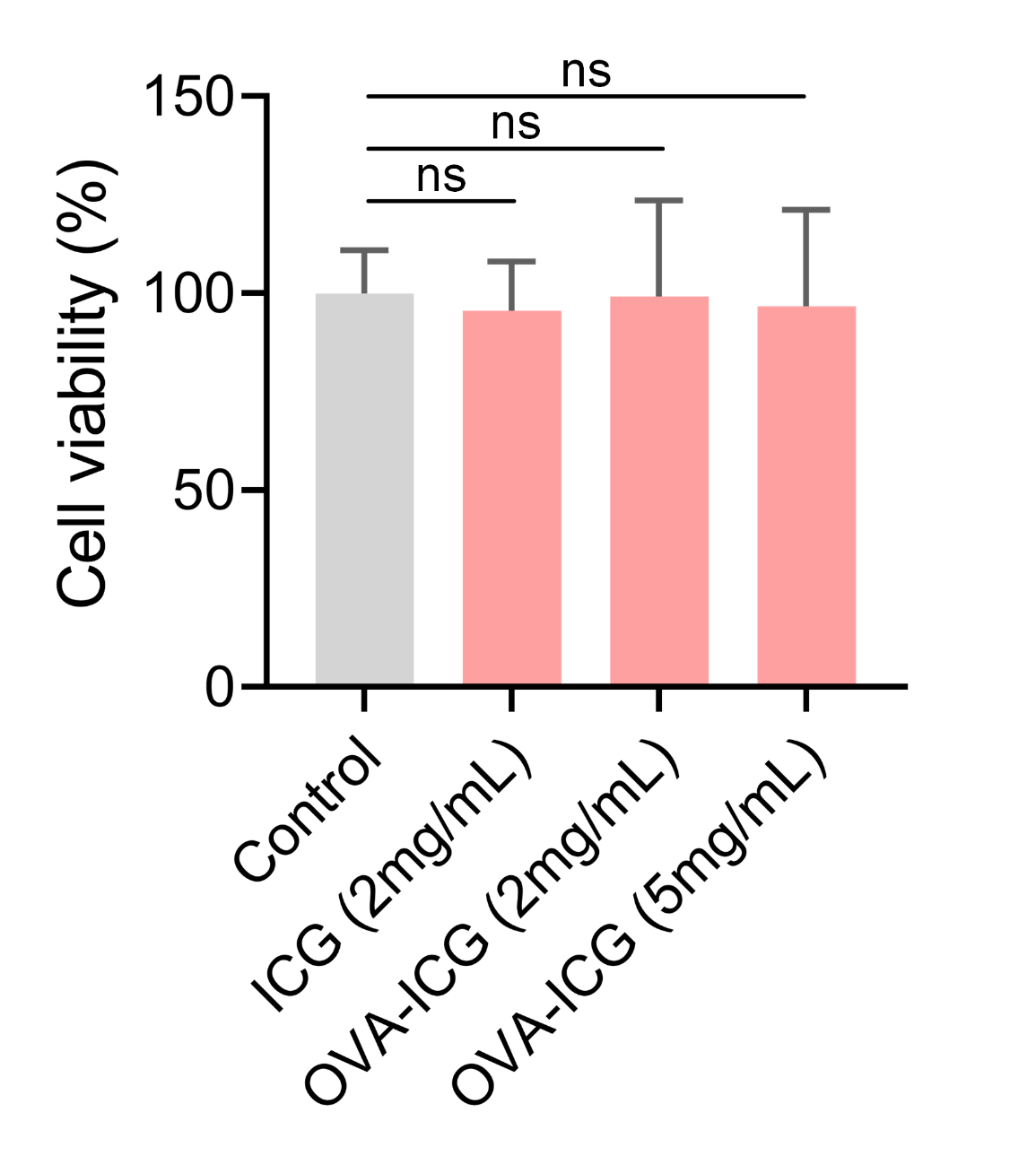


Fig. S7.

***In vitro* toxicity evaluation.** Meningeal lymphatic endothelial cells upon incubation with ICG and OVA-ICG for 24 h.


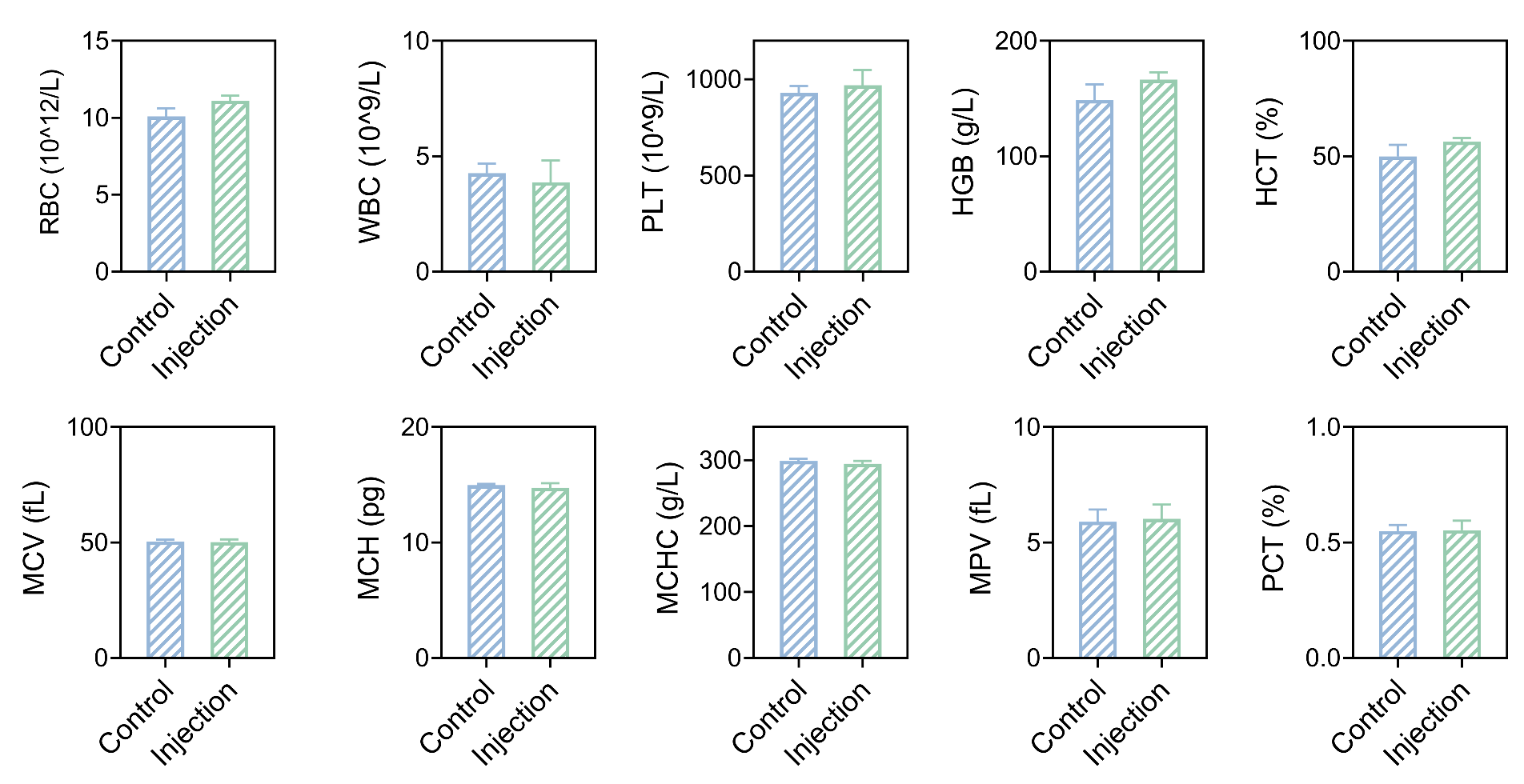


Fig. S8.

***In vivo* toxicity evaluation.** Blood hematology analyses of healthy C57 mice after cisterna magna injection with PBS and OVA-ICG for 48 hours, respectively. The terms are noted as follows: red blood cells (RBC), white blood cells (WBC), platelets (PLT), hemoglobin (HGB), hematocrit (HCT), mean corpuscular volume (MCV), mean corpuscular hemoglobin (MCH), mean corpuscular hemoglobin concentration (MCHC), and mean platelet volume (MPV), and platelet count (PCT).


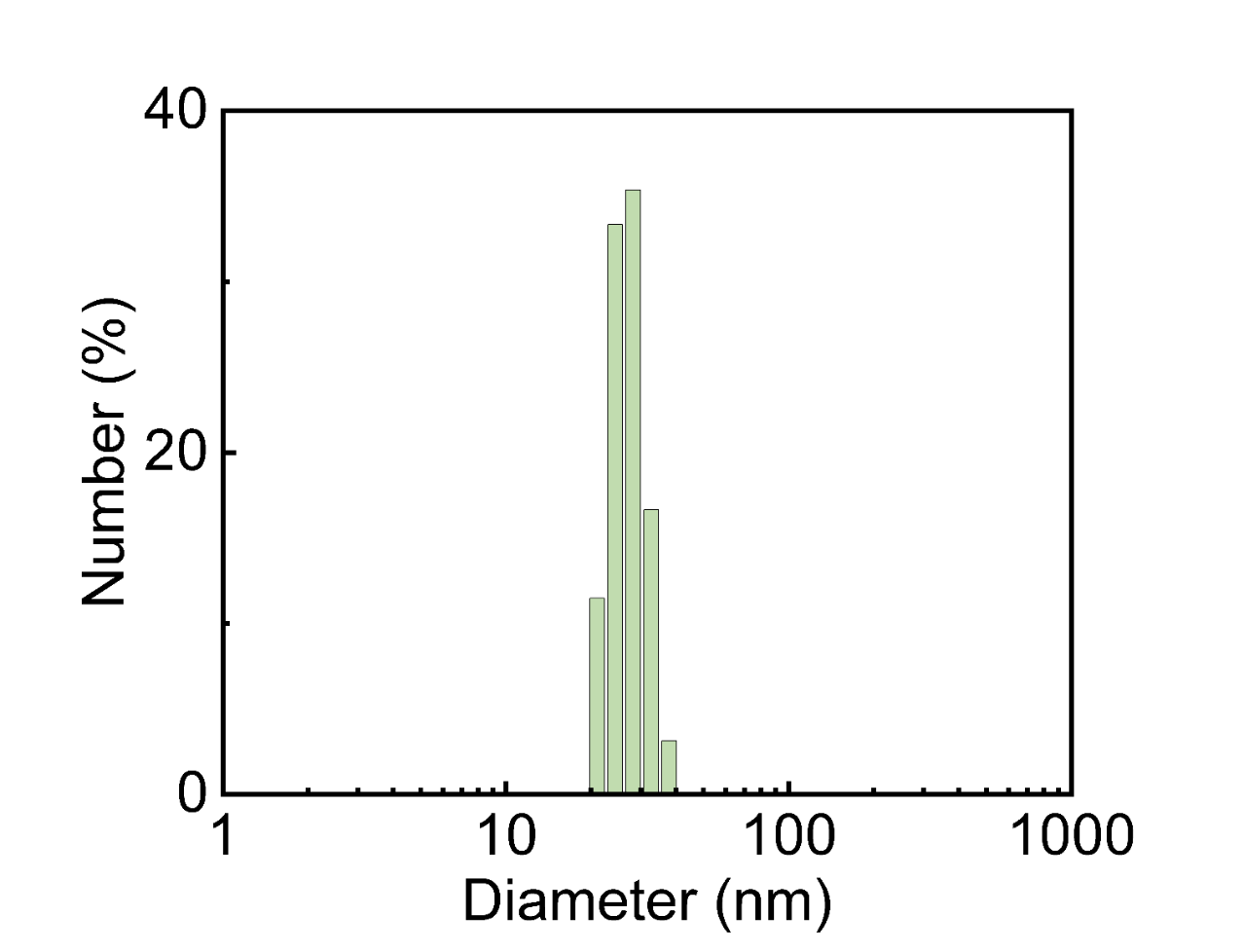


Fig. S9.

**Characterization of OVA-ICG.** The size of the synthesized OVA-ICG is verified with an average diameter of 30 nm by dynamic light scattering.


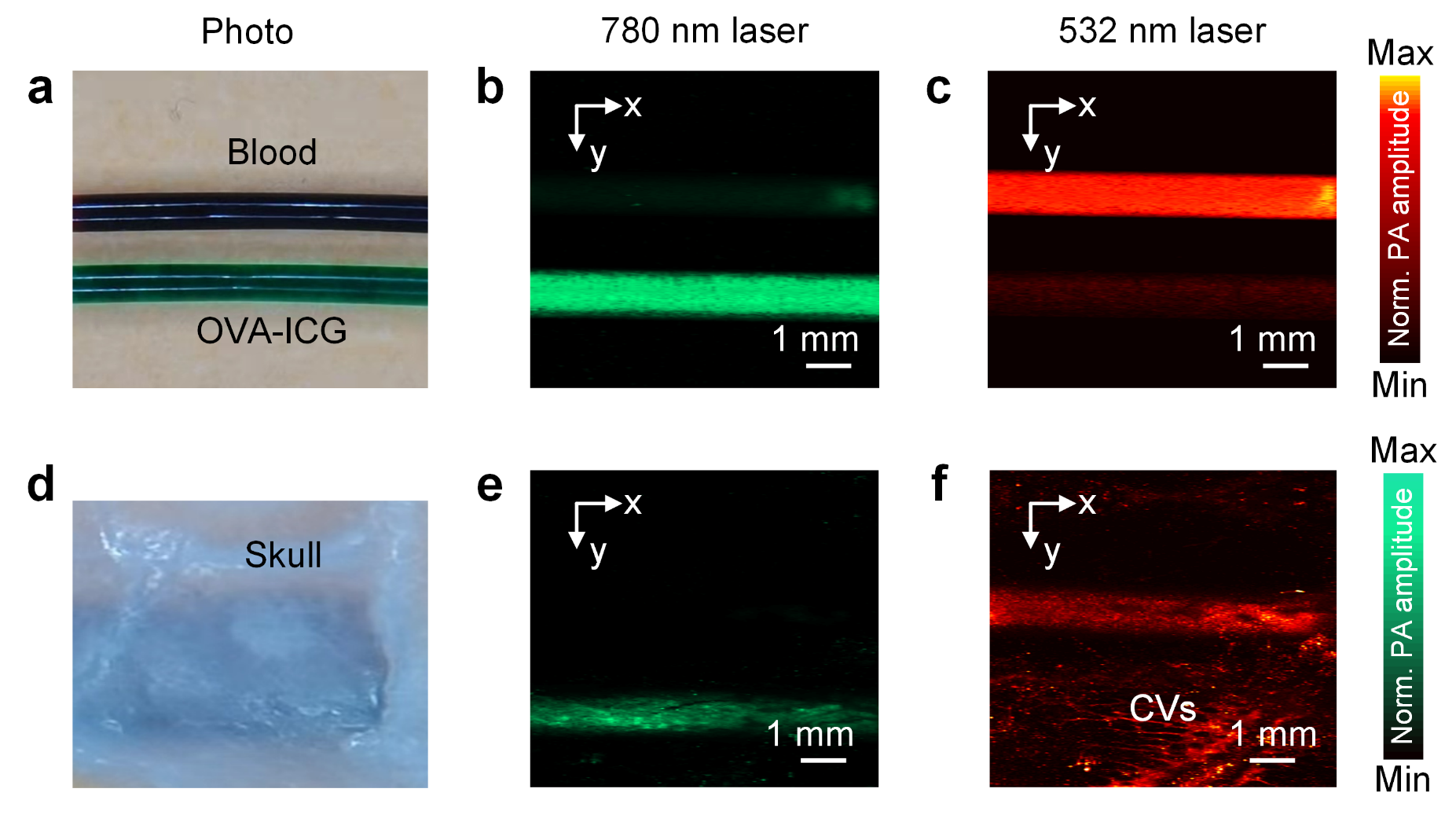


Fig. S10.

**The visible light beam (532 nm) and NIR light beam (780 nm) for photoacoustic excitation.** **a** Photograph of blood and OVA-ICG. **b** The MAP of blood and OVA-ICG using a 780 nm laser. **c** The MAP of blood and OVA-ICG using a 532 nm laser. **d** Photograph of blood and OVA-ICG under the skull. **e** The MAP of blood and OVA-ICG using a 780 nm laser under the skull. **f** The MAP of blood and OVA-ICG using a 532 nm laser under the skull. The results demonstrate that the laser energy can still excite the photoacoustic signal after through the skull.


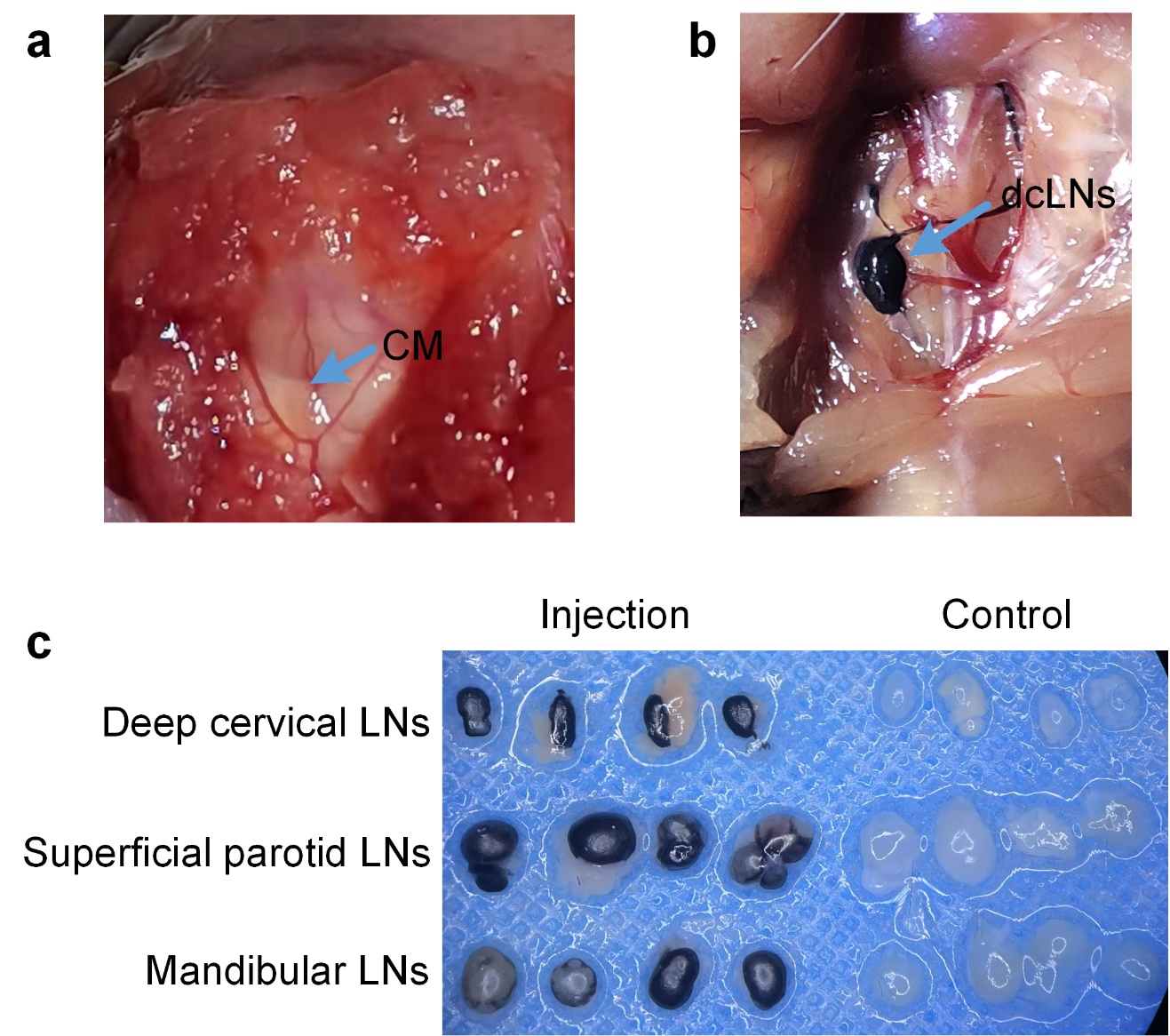


Fig. S11.

**Tracers in CSF are specifically drained by the mLVs to cLNs.** Tracers injected into the brain or CSF are found in cLNs, indicating the drainage function by mLVs ^74,75^. **a** To explore whether the tracer released into the CSF are drained to cLNs via the mLVs, we injected black ink into the mouse’s CM. **b**, **c** Two hours later, we observed that dcLNs, superficial parotid LNs and mandibular LNs were infused with ink, while no evidence of tracer was observed in the control groups. LNs, lymph nodes; dcLNs, deep cervical lymph nodes.


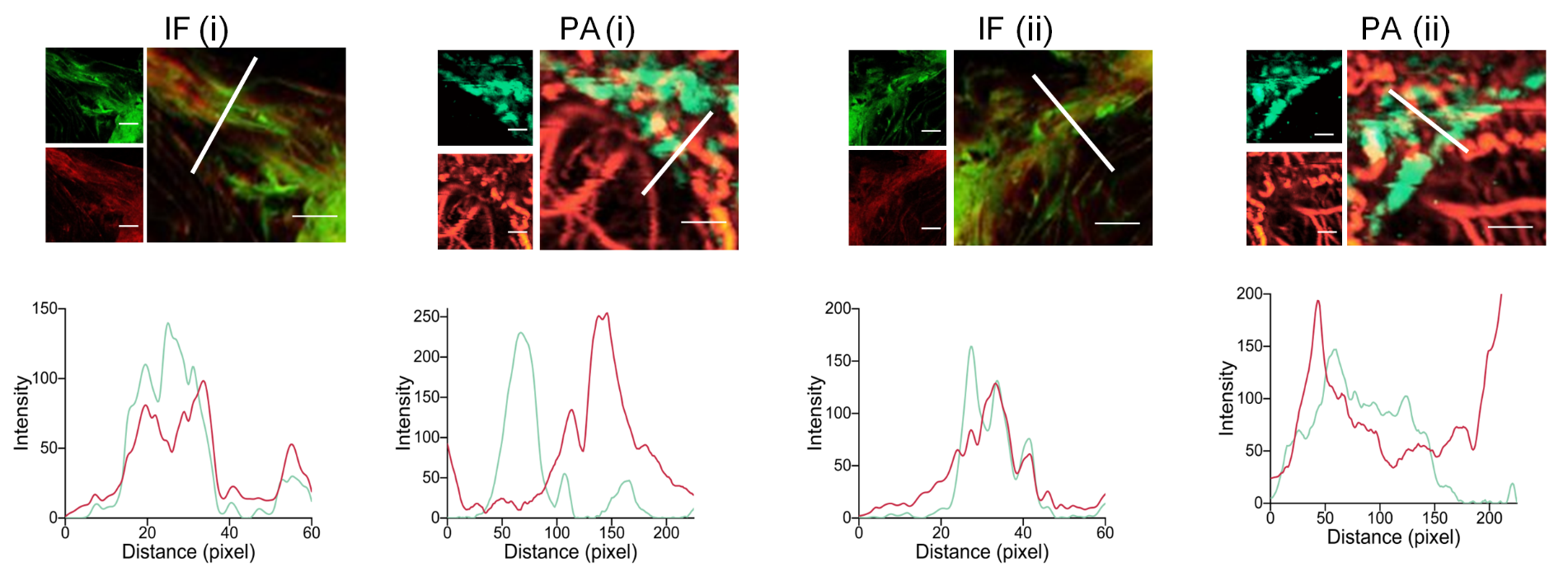


Fig. S12.

**The Representative images and co-localization analysis.** The mLVs and CVs images with immunohistochemistry, photoacoustic imaging, and merged images are acquired in different regions. And the image co-localization analysis at the pixel level of merged images. The mLVs and CVs merged images of PA modality are clearly differentiated. These PA merge images more clearly differentiate the unique spatial distribution. Scale bar, 500 μm.


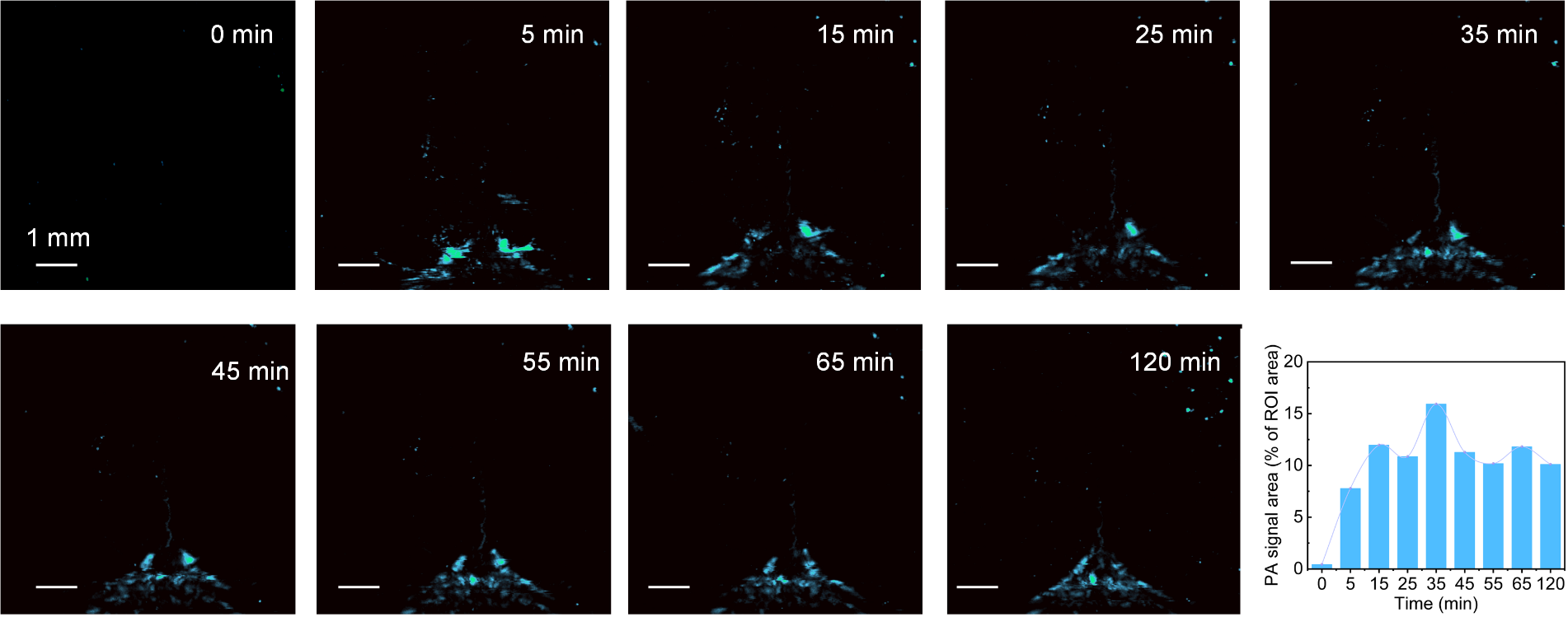


Fig. S13.

***In vivo* functional imaging of mLVs.** The MAP images before injection and 5, 15, 25, 45, 55, and 120 min after injection. And the quantitative analysis of the PA signal area of the tracer.


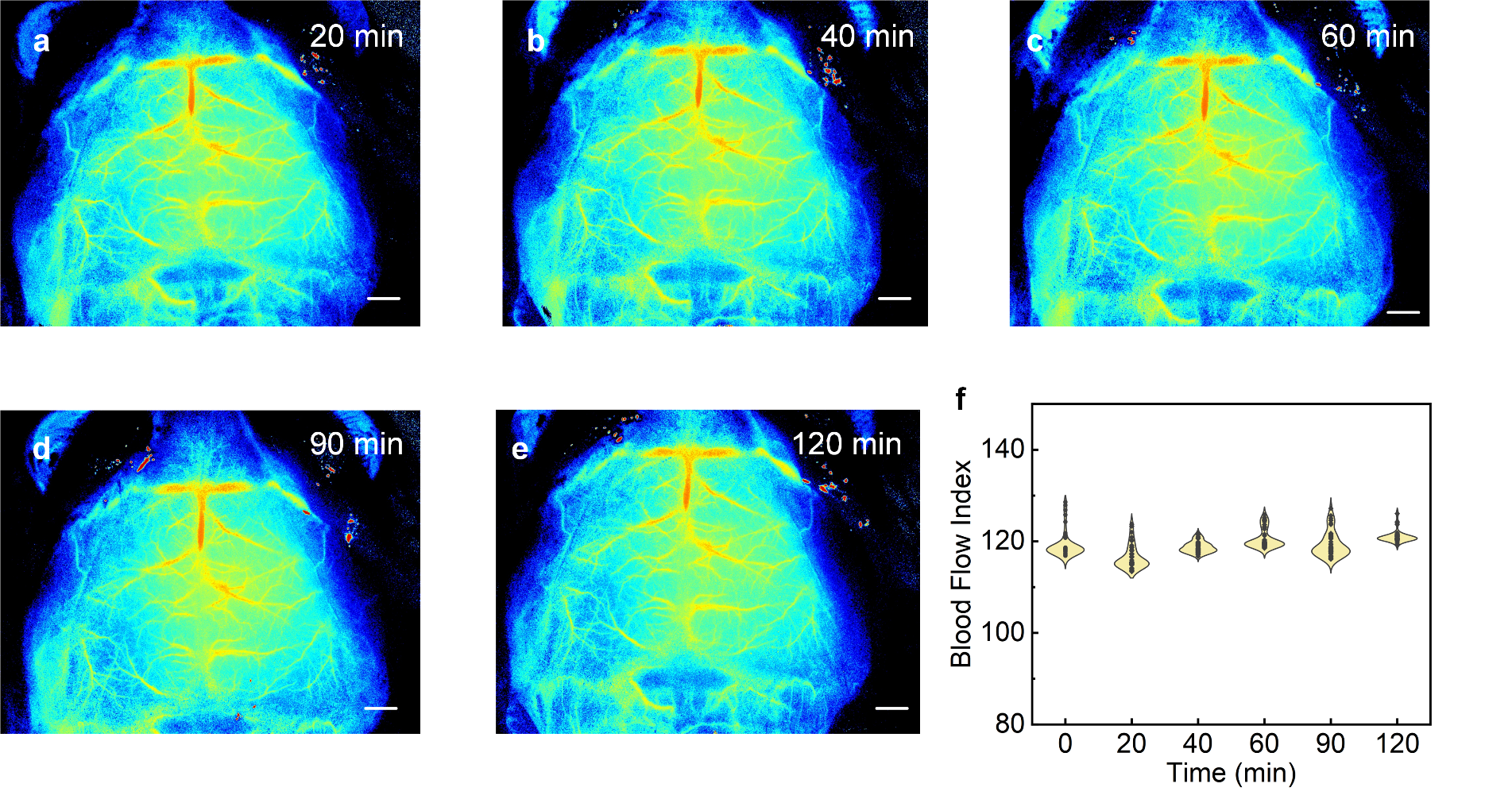


Fig. S14.

**The injection and drainage of mLVs did not alter cerebral blood flow**. **a** to **e** Representative images of cerebral blood flow detected by laser speckle. The mice are detected before injection and after injection 20 min, 40 min, 60 min, 90 min, and 120 min, respectively. Scale bar, 2 mm. **f** Quantification of the cerebral blood flow index on TS and superior sagittal sinus (SSS) (*n* = 3).


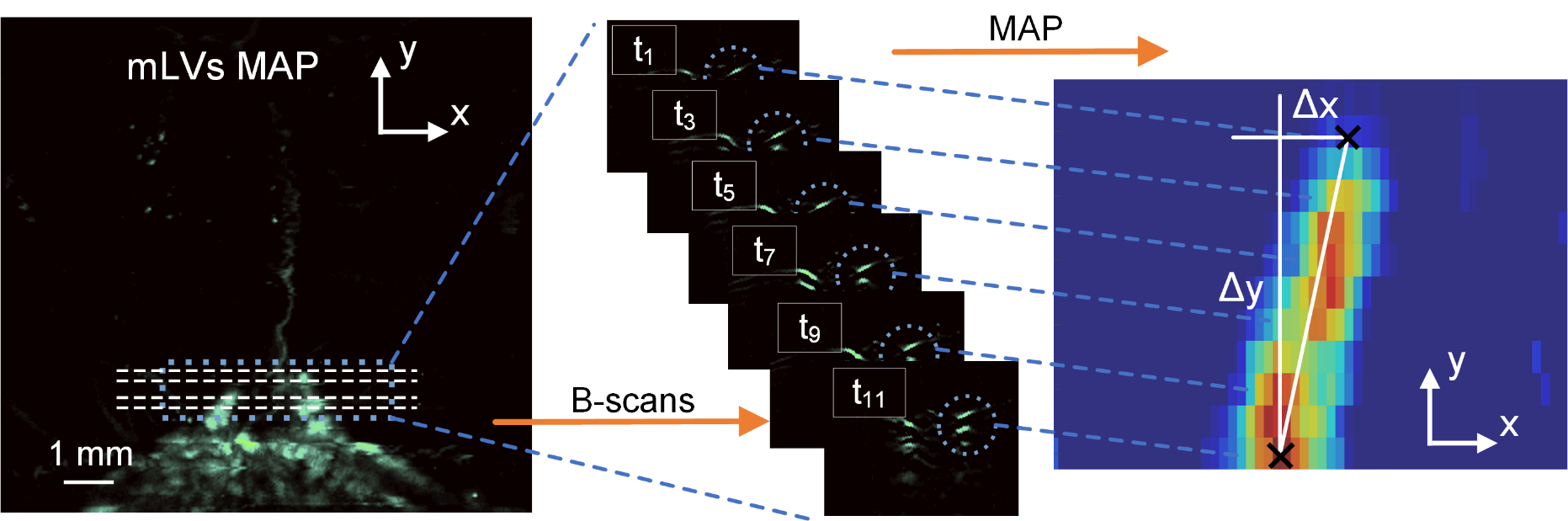


Fig. S15.

**The assessment of flow direction**. The main steps: First, a sequence of PA volume data is obtained, and the position changes of PA signals in consecutive B-scan images are tracked. Then, the maximum amplitude in a sequence of B-scan images is calculated to obtain the maximum projection on the X-Y plane. Finally, in the maximum projection image, the coordinates of the center pixel of the photoacoustic signal are taken to calculate △x and △y of the start frame and the end frame, and the movement angle = arctan (△x/△y).


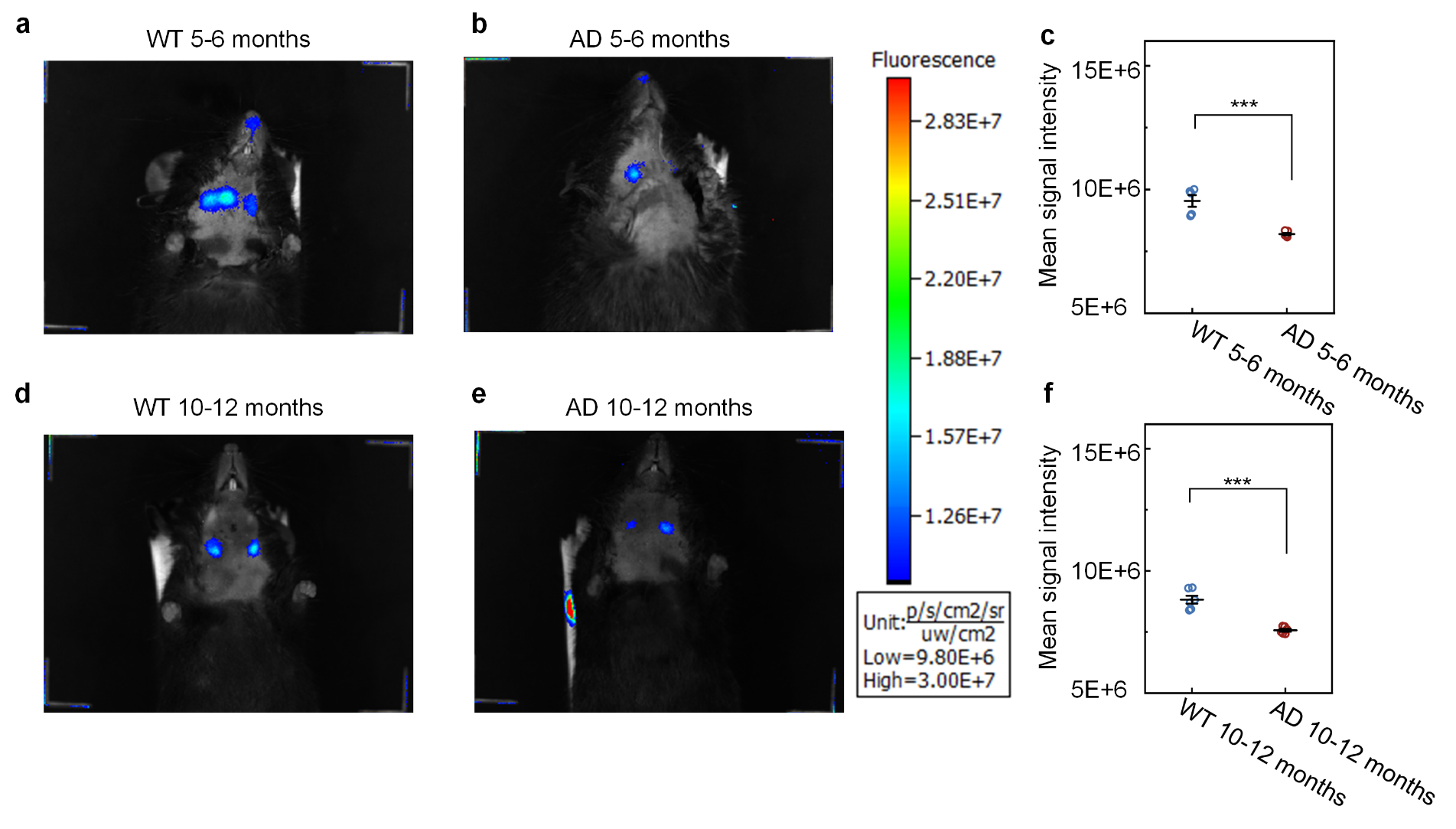


Fig. S16.

**The cLNs images of live mice are captured at 20-40min after the administration of OVA-ICG**. Five microliters of OVA-ICG (2 μg μl^-1^) are injected in the i.c.m. of WT or AD transgenic mice at 20-40 min. **a** and **b** cLNs images show the biodistribution of OVA-ICG at 5-6 months of age in WT mice and AD transgenic mice. **d** and **e** cLNs images show the biodistribution of OVA-ICG at 10-12 months of age in WT and AD transgenic mice. **c** and **f** Fluorescence accumulation of OVA-ICG is detected *in vivo* at 5-6 months and 10-12 months of age in WT and AD transgenic mice after injection (*n* =  3). Data are presented as mean ± s.e.m.; ***P*< 0.01 and ****P*< 0.001 using a two-tailed unpaired Student’s t-test.


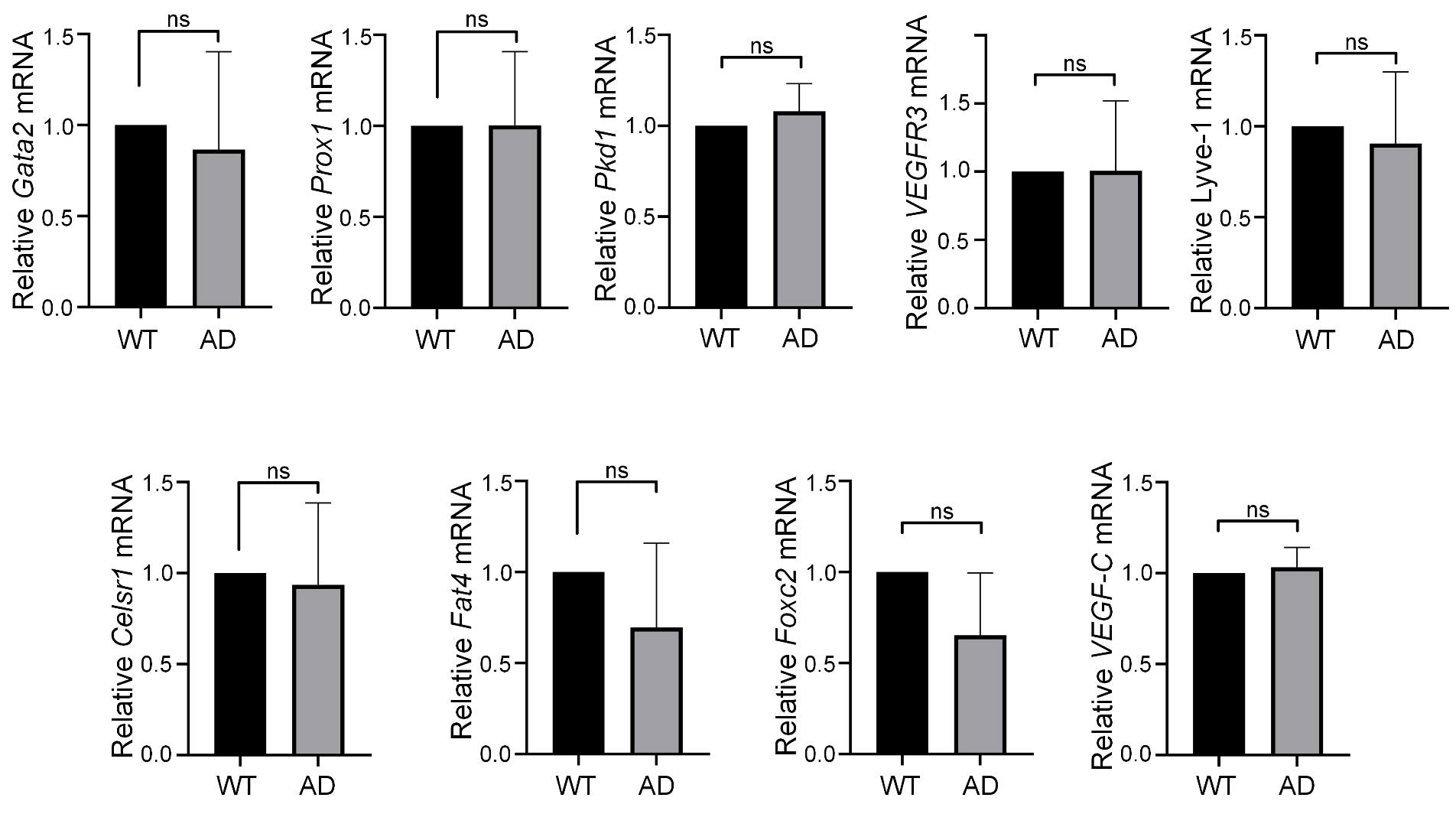


Fig. S17.

**The expression of genes relative to lymphatic vessel structure and function in mLVs of AD mice.** The relative mRNA levels of *Gata2*, *Celsr1*, *Prox1*, *Fat4*, *Pkd1*, *Foxc2*, *VEGFR3*, *Lyve-1*, and *VEGF-C* in WT or AD mice meninges. The data are presented as Mean ± SEM.

Table S1.

**PA amplitudes for different concentrations of OVA-ICG**.

| Concentration (μM) | PA amplitude |
| --- | --- |
| 10 | 0.114 ± 0.0002 |
| 20 | 0.140 ± 0.0002 |
| 30 | 0.155 ± 0.0004 |
| 40 | 0.185 ± 0.0005 |
| 50 | 0.214 ± 0.0004 |

Data are presented as mean ± s.e.m.

Table S2.

**Ratio of mLVs area to CVs area.**

| Area | Cerebrovascular area of mLVs area |
| --- | --- |
| PA-TS-I | 2.21 ± 0.02 |
| IF-TS-I | 1.48 ± 0.02 |
| PA-TS-II | 2.31 ± 0.07 |
| IF-TS-II | 1.60 ± 0.05 |
| PA-SSS | 3.65 ± 0.18 |
| IF-SSS | 1.60 ± 0.08 |

Data are presented as mean ± s.e.m.

Table S3.

**Mean PA intensity of mLVs and CVs, and fluorescence intensity of cLNs**.

| Time (min) | Mean PA Intensity | | Light Emission  (cLNs) |
| --- | --- | --- | --- |
|  | mLVs | CVs |  |
| 20 | 143.82 ± 1.58 | 60.39 ± 2.81 | 252.55 ± 0.61 |
| 40 | 134.94 ± 2.41 | 62.79 ± 2.42 | 254.37 ± 1.05 |
| 60 | 126.89 ± 2.30 | 63.11 ± 2.53 | 250.75 ± 0.38 |
| 90 | 99.30 ± 3.63 | 63.96 ± 2.79 | 251.06 ± 0.26 |
| 120 | 91.79 ± 4.10 | 61.48 ± 4.28 | 247.27 ± 0.35 |

Data are presented as mean ± s.e.m.

Table S4.

**Drainage volume by mLVs and CVs between WT 5-6 months and AD 5-6 months mice.**

| Area | | | Drainage Volume (%) | | *p* value |
| --- | --- | --- | --- | --- | --- |
|  |  |  | WT 5-6 months | AD 5-6 months |  |
| SSS | mLVs | | 50.73 ± 1.56 | 16.26 ± 1.02 | <0.0001 |
|  | CVs | | 45.95 ± 2.67 | 46.68 ± 5.25 | 0.7704 |
| TS/COS | | mLVs | 60.57 ± 4.71 | 13.70 ± 2.33 | <0.0001 |
|  |  | CVs | 41.67 ± 4.37 | 45.60 ± 2.89 | 0.0964 |

Data are presented as mean ± s.e.m., two-tailed unpaired Student’s t-test.

Table S5.

**Drainage volume by mLVs and CVs between WT 10-12 months and AD 10-12 months mice.**

| Area | | | Drainage Volume (%) | | *p* value |
| --- | --- | --- | --- | --- | --- |
|  |  |  | WT 10-12 months | AD 10-12 months |  |
| SSS | mLVs | | 54.54 ± 4.14 | 13.76 ± 4.31 | <0.0001 |
|  | CVs | | 50.05 ± 1.88 | 43.96 ± 3.31 | 0.0029 |
| TS/COS | | mLVs | 55.88 ± 4.14 | 12.41 ± 1.30 | <0.0001 |
|  |  | CVs | 47.31 ± 2.15 | 45.82 ± 3.26 | 0.3735 |

Data are presented as mean ± s.e.m., two-tailed unpaired Student’s t-test.

Table S6.

**Mean intensity by mLVs between 5-6 months and 10-12 months mice.**

| Group | | | Mean intensity (% ROI) | | *p* value | |
| --- | --- | --- | --- | --- | --- | --- |
|  |  |  | WT | AD |  |  |
| SSS | | 5-6 months | 16.92 ± 0.66 | 15.91 ± 1.20 | | 0.2694 |
|  |  | 10-12 months | 18.45 ± 1.22 | 11.26 ± 1.34 | | 0.0023 |
| TS/COS | 5-6 months | | 19.27 ± 0.62 | 19.72 ± 0.25 | | 0.3155 |
|  | 10-12 months | | 13.63 ± 0.99 | 11.20 ± 1.13 | | 0.0485 |

Data are presented as mean ± s.e.m., two-tailed unpaired Student’s t-test.

Table S7.

**Drainage volume by mLVs and CVs between young mice (WT 1-2 months) and adult mice (WT 5-6 months and WT 10-12 months).**

| Area | | Drainage Volume (%) | | *p* value |
| --- | --- | --- | --- | --- |
|  |  | Young | Adult |  |
| SSS | mLVs | 3.90 ± 3.14 | 23.68 ± 8.46 | 0.0012 |
|  | CVs | 28.71 ± 12.88 | 24.28 ± 15.11 | 0.6310 |
| TS/COS | mLVs | 12.27 ± 5.37 | 27.89 ± 9.11 | 0.0108 |
|  | CVs | 22.86 ± 8.87 | 21.65 ± 7.76 | 0.8245 |

Data are presented as mean ± s.e.m., two-tailed unpaired Student’s t-test.

Table S8.

**Quantitative RT-PCR primers**.

| ***Gene*** | ***Species*** | **Forward primer (5’-3’)** | **Reverse primer (5’-3’)** |
| --- | --- | --- | --- |
| *Gata2* | Mouse | CACCCCGCCGTATTGAATG | CCTGCGAGTCGAGATGGTTG |
| *Celsr1* | Mouse | TCGCTGACTTCGGTGCTTG | TTACCAGCTCTACCCAAACGG |
| *Fat4* | Mouse | CAGTGGTGATCCAGGTACGG | TCATGCGCTGTCACGGAAATA |
| *Focx2* | Mouse | AACCCAACAGCAAACTTTCCC | GCGTAGCTCGATAGGGCAG |
| *Prox1* | Mouse | AGAAGGGTTGACATTGGAGTGA | TGCGTGTTGCACCACAGAATA |
| *Sema3a* | Mouse | GGCTGGTTCACTGGGATTG | CCGTTTGCATAGTTTGCTCTGG |
| *Egfl7* | Mouse | CTGCTTGTAGCATGGTTTCTAGT | TACGGCTGGGTCTGTAGACAT |
| *Nrp1* | Mouse | GACAAATGTGGCGGGACCATA | TGGATTAGCCATTCACACTTCTC |
| *Pkd1* | Mouse | CTAGACCTGTCCCACAACCTA | GCAAACACGCCTTCTTCTAATGT |
| *VEGFR3* | Mouse | ACCTCTCCAACTTCTTGCGTGTCA | GGTGCTTCCAAGTCTCCTCCTATCA |
| *Lyve-1* | Mouse | AGGAGCCCTCTCCTTACTGC | ACCTGGAAGCCTGTCTCTGA |
| *VEGF-C* | Mouse | AGCTGAGGTTTTTCTCTTGTGATTTAA | TGATCACAGTGAGCTTTACCAATTG |

References.

73. J. Gao, Q. Li, H. Zhao, L. Li, C. Liu, Q. Gong, L. Qi, One-pot synthesis of uniform Cu_2_O and CuS hollow spheres and their optical limiting properties. *Chemistry of Materials* **20**, 6263-6269 (2008).

74. Q. Ma, B. V. Ineichen, M. Detmar, S. T. Proulx, Outflow of cerebrospinal fluid is predominantly through lymphatic vessels and is reduced in aged mice. *Nat Commun* **8**, 1434 (2017).

75. W. Van den Broeck, A. Derore, P. Simoens, Anatomy and nomenclature of murine lymph nodes: Descriptive study and nomenclatory standardization in BALB/cAnNCrl mice. *J Immunol Methods* **312**, 12-19 (2006).

Movie S1.

The stereoscopic PA imaging of mLVs, glymphatic pathways, and cerebral vessel.

Movie S2.

The flow direction of the tracer.
